# Systematic review of the subcutaneous air pouch model using monosodium urate and calcium pyrophosphate and recommendations for studying crystal‐related arthropathies

**DOI:** 10.1002/ame2.70058

**Published:** 2025-07-11

**Authors:** Wenu Hewage, Josif Vidimce, Ryan G. Shiels, Michael Morgan, Andrew C. Bulmer

**Affiliations:** ^1^ School of Pharmacy and Medical Science Griffith University Gold Coast Queensland Australia; ^2^ School of Medicine and Dentistry Griffith University Gold Coast Queensland Australia; ^3^ Queensland Alliance for Environmental Health Science University of Queensland Brisbane Queensland Australia; ^4^ Department of Anatomy and Physiology University of Melbourne Melbourne Victoria Australia

**Keywords:** arthritis therapeutics, immunology, inflammatory mediators, joint injury, murine model, rheumatic and musculoskeletal disease

## Abstract

The subcutaneous air pouch model has been used extensively to study the pathophysiology of inflammatory conditions such as joint diseases and the potential efficacy of pharmacological treatments in vivo. Delivery of air between the subcutaneous and dermal layer of the intra‐scapular zone of the rodent generates an environment analogous to the synovial joint space. Introduction of monosodium urate crystals or calcium pyrophosphate crystals into the air space produces a sterile acute inflammatory response mimicking clinical gout and pseudogout, respectively. The inflammatory response can be quantitatively and robustly evaluated by measuring leukocyte infiltration, inflammatory cytokine production, eicosanoid release, complement activation and reactive oxygen species generation. Despite the utility of this model, great variation exists within the literature regarding the design, sampling time points, and endpoints measured. This systematic review summarizes the current literature on the subcutaneous air pouch model studying monosodium urate or calcium pyrophosphate crystals and provides recommendations for standardizing and improving the reliability and validity of this model. Standardizing the experimental approach would improve inter‐study comparability, increase the internal validity of studies and reproducibility of results, and ultimately improve the understanding of gout and pseudogout and accelerate the discovery of new pharmacological therapies.

## Graphical abstract

Simplified schematic for generation of the subcutaneous air pouch model in the rodent. On day 0 and 4, air is injected into the subcutaneous space on the dorsal surface of the rodent to generate a cavity analogous to a synovial joint. On day 6, monosodium urate (MSU) or calcium pyrophosphate (CPP) crystals are injected into the pouch to stimulate sterile inflammation as observed in gout and pseudogout, respectively. Animals are pre‐treated with potential anti‐inflammatory agents based on their pharmacokinetic profile such that their peak serum concentrations align with stimulation of inflammation. The pouch fluid is consecutively aspirated at desired time points after stimulation of inflammation for analysis of inflammatory‐cell infiltration and mediator concentrations. After the final collection, animals are euthanised and the pouch membrane is dissected for histological assessment of inflammation. Leukocyte infiltration and inflammatory mediator concentrations in the pouch fluid, and histological changes in pouch tissue are compared to assess changes in the inflammatory response and the efficacy of therapeutic interventions. Graphical abstract created using biorender.com.

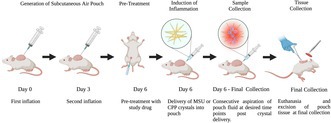

## 1 INTRODUCTION

Gout and pseudogout are caused by an inflammatory reaction to intra‐articular deposition of monosodium urate (MSU) and calcium pyrophosphate (CPP) crystals within the synovial joint, respectively, with the former being the most prevalent form of inflammatory arthritis.^1^ These conditions are characterized as subtypes of chronic inflammatory arthritis that are interposed by cyclical attacks of acute inflammatory attacks.^2^, ^3^ Consequently, there is progressive joint degeneration that can result in significant functional impairment; therefore, they are a significant health and economic burden globally. Current treatments are ineffective at preventing disease progression; thus, novel therapeutics are urgently required.^4^

Gout is commonly observed in individuals with elevated serum uric acid, an end‐product of purine metabolism.^3^, ^5^ The epiphyses of the synovial joint are enclosed by articular cartilage and a thin membrane of synoviocytes that are specialized macrophages and fibroblast‐like cells. In a multi‐step process, when serum or local uric acid concentrations exceed their solubility limits, urate anions within the synovial environment associate with water and sodium to form MSU crystals. Factors including local temperature, pH, mechanical stress and tissue components are hypothesized to promote crystal formation.^5^ In contrast, pseudogout is caused by imbalance between pyrophosphate metabolism and the level of pyrophosphatases in diseased cartilage, and is often associated with aging.^6^ As pyrophosphate deposits in the synovium and adjacent tissues, calcium combines with it to form calcium pyrophosphate. Calcium pyrophosphate (CPP) crystals consist of monoclinic and triclinic phases (m‐CPP and t‐CPP, respectively), with the former shown to play a more active role.^7^ Interestingly, the overlapping inflammatory mechanisms and clinical presentations of gout and pseudogout often lead to diagnostic challenges. Definitive diagnosis of crystal‐induced arthritis is demonstrated by a direct examination of crystals in synovial fluid aspirates. Monosodium urate crystals are needle‐shaped and show strong negative birefringence, whereas CPP crystals are rhomboid shaped and have no or weak positive birefringence.^8^

Consistent with sterile acute inflammation, MSU and CPP crystals initiate, amplify, and sustain an intense inflammatory response. Upon contact with resident cells, MSU crystals destabilize the cell membrane.^1^ Similarly, CPP crystals directly interact with and activate synovial resident cells.^7^ Both are believed to trigger events that lead to phagocytosis and cytokine release predominantly through the nucleotide binding domain, leucine‐rich‐containing family, nod‐like receptor family pyrin domain containing‐3 (NLRP3) inflammasome. This induces the production of interleukin (IL)‐1β and accompanying full spectrum of sterile inflammation leading to significant hyperplasia of the lining membrane and infiltration with leukocytes consisting of neutrophils, monocytes, and lymphocytes.^1^, ^9^ For this reason therapeutics that target IL‐1β, including anakinra and canakinumab, are used as second‐line treatments for acute gout flare‐ups in patients who are intolerant of or have contraindications for first line, symptom modifying corticosteroids and non‐steroidal anti‐inflammatory drugs (NSAIDs).^10^ Unlike gout, where therapies targeting uric acid metabolism exist, current treatments for CPPD are symptom‐based and ineffective in preventing disease progression, underscoring the need for novel therapeutics. Release of other inflammatory cytokines including tumor necrosis factor (TNF)‐α, IL‐6 and eicosanoids (e.g. prostaglandins) are also observed in both conditions.^1^, ^7^, ^11^, ^12^, ^13^ Monosodium urate crystals have also been shown to activate the complement cascade and induce production of reactive oxygen spices (ROS).^1^, ^13^, ^14^, ^15^, ^16^, ^17^, ^18^ For this reason, complement inhibition and antioxidant agents may offer novel targets in the treatment of gout.

The subcutaneous air pouch is an in vivo animal model that studies components of the inflammatory response to disease associated molecular pattern (DAMPS; such as MSU and CPP crystals) and pathogen associated molecular pattern (PAMPs; such as carrageenan) molecules and how they change in the presence of therapeutic agents.^19^ More recently, the model has been adapted in oncological studies evaluating tumor progression and treatment efficacy.^20^ The model was first described by Selye,^21^ who injected air between the subcutaneous and dermal layer of the intra‐scapular zone in rodents to generate an avascular cavity, henceforth referred to as a ‘pouch’. Over 3–4 days, the pouch wall becomes progressively lined with mononuclear cells and fibroblast‐like cells, analogous to synoviocytes. These fibroblast‐like cells secrete collagen, leading to thickening of the pouch wall and subsequent vascularisation of the pouch cavity. This cavity also demonstrates key characteristics of the synovial lining, including the synthesis of hyaluronic acid.^22^ This combination of collagen deposition, new blood vessel formation and production of synovial compounds creates an environment analogous to the synovial joint. For this reason, the subcutaneous air pouch model is most suited to studying mechanisms and treatment of inflammatory pathways triggered by DAMPs and PAMPs that commonly occur within the synovial cavity, such as gout and pseudogout. Delivery of other agents such as carrageenan to stimulate inflammation in the subcutaneous air pouch have also been described in the literature and are reviewed elsewhere. Additional air is delivered to maintain the patency of the cavity.^23^ Once a patent cavity is established, insoluble MSU and CPP crystals are injected into the pouch, inducing inflammatory exudate formation with leukocyte infiltration, production of pro‐inflammatory cytokines and oxidative stress as observed in inflammatory arthropathies.^24^ The inflammatory exudate or ‘pouch fluid’ can be collected via aspiration of the cavity or by blunt dissection of the pouch tissue. This fluid can then be analyzed to quantify the inflammatory response via characterizing inflammatory cell populations, cytokine production, eicosanoid release, oxidative damage and biochemical markers of inflammation. The pouch tissue can also be isolated and analyzed for inflammatory protein and gene expression along with immunohistochemical and histopathological tissue changes. Importantly, potential therapeutic targets can be tested to inform their anti‐inflammatory efficacy. The acute inflammatory response can also be studied over time via serial sampling of pouch fluid. Therefore, the temporality of inflammation resolution can be assessed which includes assessment of counter‐regulatory anti‐inflammatory mechanisms, in addition to inhibition of pro‐inflammatory stimuli and related mechanisms. Studies investigating MSU and CPP crystal‐induced inflammation in this model are summarized in Tables S1 and S2, respectively, in supporting information. The parameters that have been measured within these studies are summarized in Table 1. The model also has the advantage of being less technically challenging and more robust compared to other models, including those involving delicate procedures on animal joints.^25^ Moreover, from an ethical perspective, there is reduced burden on animals as inflammation in the pouch model is localized within the cavity and therefore painless, and does not trigger a systemic immune response.^25^ In models involving injection of MSU crystals into weight bearing joints, animals experience significant pain.^26^ On these bases, the subcutaneous pouch model is relatively easy to conduct and reproducible in terms of inflammatory response, which will be further discussed throughout this review. Furthermore, the model effectively mirrors key inflammatory mechanism observed in human crystal arthropathies, offering valuable insights into disease pathophysiology. Both exhibit neutrophil‐driven inflammation, robust IL‐1β production, and activation of the NLRP3 inflammasome in response to crystals. Therefore, the air pouch model represents a highly relevant preclinical tool for studying the shared molecular and cellular pathways involved in crystal associated inflammation. Moreover, it permits testing of therapies for which limited, well‐tolerated treatment options currently exist. Although protocols for the generation of the model have been previously described,^18^ to the authors' knowledge, no systematic review on crystal related inflammation in the air pouch model has been previously published and therefore conclusions of this review are key to guiding future application of the model in research. This systematic review summarizes the varying approaches to using the air pouch model to study crystal related arthropathies. Furthermore, it recommends a standardized model to enable direct comparisons between results in the literature. Options are also specified to provide researchers with a means to optimize data collection and choose from relevant endpoints.

**TABLE 1:** Inflammatory mediators related to monosodium urate and calcium pyrophosphate crystal inflammation in the air pouch model.

Inflammatory mediator	Analyte within pouch fluid
Cytokines	IL‐1Ra ^27^ IL‐1β* ^7^, ^27^, ^28^, ^29^, ^30^, ^31^, ^32^, ^33^, ^34^, ^35^, ^36^, ^37^, ^38^, ^39^, ^40^, ^41^, ^42^, ^43^, ^44^, ^45^, ^46^ IL‐4 ^33^ IL‐6* ^27^, ^28^, ^31^, ^33^, ^36^, ^40^, ^42^, ^43^, ^44^, ^47^, ^48^, ^49^, ^50^, ^51^, ^52^, ^53^, ^54^ IL‐10 ^55^ IL‐18* ^30^, ^32^, ^37^, ^45^, ^56^ TNF‐α* ^27^, ^33^, ^37^, ^43^, ^44^, ^45^, ^46^, ^49^, ^51^, ^53^, ^54^, ^55^, ^57^ CCL2* ^27^, ^41^, ^42^, ^53^, ^58^ CCL3 ^58^ CXCL1/KC* ^7^, ^34^, ^39^, ^41^, ^42^, ^49^, ^52^, ^58^ CXCL2 ^42^, ^58^ MIP‐1α ^49^ MIP‐2 ^31^, ^48^ MCP‐1* ^31^, ^36^, ^44^, ^56^ GM‐CSF ^56^
Eicosanoids	PGE2* ^51^, ^57^, ^59^ PGD2 ^51^ PGE2 ^60^ LTB4* ^59^, ^61^, ^62^ TXB2 ^59^ 6‐oxo‐PGF1⍺ ^59^ 6‐Keto‐PGF1⍺ ^63^
Amines	Histamine ^28^, ^64^
Oxidant enzymes, reactive oxygen/nitrogen species	MPO* ^18^, ^31^, ^32^, ^37^ Nitric oxide ^28^
Other	Caspase‐1 activity ^32^ GADPH ^35^ HIF‐1α ^35^ PPIA ^35^ Complement—C3a/C5a ^33^ NLRP3 ^38^

*Note*: Measures marked with an asterisk (*) were defined as having strong evidence in this model, as they have been widely reported (3 or more studies).

Abbreviations: CCL, chemotactic ligand; CXC, chemokine (C‐X‐C motif) ligand; GADPH, glyceraldehyde‐3‐phosphate dehydrogenase; GM‐CSF, granulocyte macrophage colony‐stimulating factor; HIF, hypoxia inducible factor; IL, interleukin; IL‐1Ra, interleukin‐1 receptor antagonist; KC, keratinocyte chemoattractant; LT, leukotriene; MCP, monocyte chemoattractant protein; MIP, macrophage inflammatory protein; MPO, myeloperoxidase; NLPR3, nucleotide‐binding oligomerization domain, leucine rich repeat and pyrin domain containing; PG, prostaglandin; PGF, platelet growth factor; PPIA Peptidylprolyl isomerase A; TNF, tumor necrosis factor; TX, thromboxane.

## 2 METHODS

### 2.1 Search strategy and study selection

The present review summarizes published studies on MSU and CPP crystal inflammation in the subcutaneous air pouch model. The PubMed database was searched using search terms presented in Table 2. The search period included studies from the databases inception to March 2025. Results were limited to those with an English title and abstract. The selection of studies for full reading and inclusion in the review were made through reading of abstracts. Studies were excluded if they did not investigate MSU or CPP crystal inflammation or did not perform the subcutaneous air pouch model. In total, 83 articles were selected for this review and are summarized in Tables S1 and S2 in supporting information. Of the reviewed articles, 75 studies investigated MSU crystals exclusively, two focused solely on CPP crystals, and six examined both crystal types.

**TABLE 2:** Literature search.

Search terms
(Air OR subcutaneous OR rat OR murine) AND (Pouch) AND (Urate OR monosodium OR MSU OR uric acid OR gout OR pseudogout OR CPP OR calcium pyrophosphate)

### 2.2 Data extraction

The extracted data from the studies included the first author, method of crystal procurement, animal characteristics (sex, species, strain, age and sample size), pouch generation (volume of air used and number of inflations), induction of inflammation (time of pouch stimulation, MSU and/or CPP crystal quantity and suspension medium and volume), pouch fluid extraction (harvesting technique, medium and volume and collection time points), sample handling and measured outcomes (leukocyte counts, inflammatory mediators measured and respective concentrations).

### 2.3 Data analysis

Unless explicitly stated, recommendations are presented as median and interquartile range (IQR). Where the IQR provides no meaningful insight, it was omitted. Where studies provided only ranges, the average of the range was used, for example 4–5 sample size, the average sample size was 4.5. These values were included to calculate the overall median across studies. For qualitative data, the attribute most frequently mentioned was used. Where insufficient data was available, the authors made recommendations based on experience, which have been explicitly stated. One study investigated three different methods for the model,^24^ and was therefore analyzed as three separate studies for calculations.

## 3 DISCUSSION

### 3.1 Generation of monosodium urate and calcium pyrophosphate crystals

While some published studies have obtained MSU (InvivoGen, Cat# tlrl‐msu) and CPP (InvivoGen, Cat# tlrl‐cppd) crystals from a supplier, the majority of authors synthetised their own MSU (55 of 81, 12 not specified) and CPP crystals (4 of 8, 3 not specified) according to methods described in the literature. Discussion and comparison of different synthesis methods is beyond the scope of this review as this has been discussed elsewhere.^45^, ^65^ Instead the methods are briefly summarized below with practical recommendations.

**FIGURE 1:**
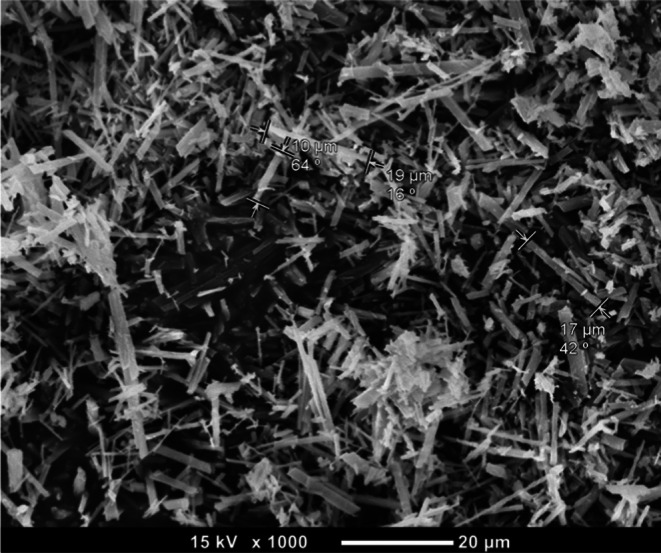
Scanning electron microscope image of the authors' MSU crystals at ×1000 magnification.

All synthesis should be performed under sterile conditions. For synthesis of MSU crystals, briefly, uric acid is dissolved in aqueous sodium hydroxide (NaOH) under heat and cooled, leading to precipitation of MSU crystals. Methods differ with respect to the temperature the solution is heated to, amount of uric acid, sodium hydroxide and sodium chloride (NaCl) added, pH of the solution (by addition of hydrochloric acid, HCl), and the subsequent rate of cooling of the solution. Differences in these factors yield crystals varying in size, morphology, surface area and ultrastructure, which in turn influences crystal phagocytosis, membranolytic effects and protein binding mediated reactions and therefore the nature of the inflammatory response elicited. For example, smaller crystals exhibit greater inflammatory potential as they are more readily phagocytosed, leading to increased activation of intracellular signaling pathways.^65^, ^66^ It is possible that variation in the MSU crystal sources and morphology may explain conflicting evidence of the molecular mechanisms underlying MSU crystal induced inflammation. Therefore, it is important to implement standardized methods in the preparation of MSU crystals. Monosodium urate crystals observed in clinical gout have a characteristic needle‐like appearance and are 8.63 ± 3.19 μm (mean ± standard deviation) in length.^65^ The method described to synthesize MSU crystals by McCarty and Mandel yields crystals that most closely resemble sizes reported clinically.^65^ If synthesis is attempted, the method can be adjusted according to the variables described in Table 3. In the authors' experience, having attempted numerous methods, they found a modified method of McCarty whereby the pH was maintained at 8.9 without addition of HCl was the easiest to apply and created crystals that were 11.4 ± 5.52 μm in length as shown in Figure 1.^56^

**TABLE 3:** Parameters for monosodium urate crystal synthesis methods.

Method	Mandel et al. ^65^	Akali titration (Denko et al.) ^65^	Acid titration (McCarty et al.) ^65^
Reagent			
Uric acid	8 g	4 g	1.68 g
ddH_2_O	1600 mL	800 mL	400 mL
NaOH	1 M, 45 mL	0.5 M, 49 mL	1 M, 6 mL
HCl	1 M, until target pH	1 M, until target pH	1 M, until target pH
Condition			
pH	8.0	8.9	7.0
Heating temperature	100°C	60°C	60°C
Stirring	Stirred at 15 RPM for 5 s upon addition of each reagent		
Storage	23°C for 24 h	23°C for 24 h	23°C for 24 h

*Note*: Summary of studies evaluating different MSU crystal synthesis methods ^45^, ^65^.

Abbreviations: ddH_2_O, double distilled water; HCl, hydrochloric acid; NaOH, sodium hydroxide; RPM, revolutions per minute.

Various methods for CPP crystal synthesis are also reported. Differences in the size, structure, surface area, shape and proportion of CPP phases could also explain differences in the clinical phenotypes of pseudogout. Pre‐clinical studies show that acute inflammatory synovial fluid contains a greater portion of the smaller and therefore more phagocytically active m‐CPP phase compared to low inflammatory samples, which contain a greater portion of the larger t‐CPP phase.^7^ Moreover, the use of m‐CPP crystals in the air pouch model induces a stronger inflammatory response, characterized by greater leukocyte infiltration and increased release of inflammatory mediators, compared to t‐CPP crystals.^7^ Therefore, it is recommended that studies utilize m‐CPP crystal preparations to evaluate the acute inflammatory response and identify potential therapeutic targets for pseudogout. Methods described by Martinon et al. also yield m‐CPP crystals that closely mimic those observed in clinical pseudogout.^11^ Briefly, calcium nitrate (Ca(NO_3_)_2_, 0.1 M final concentration) is mixed with an acidic sodium pyrophosphate (Na_2_P_2_O_7_, 25 mM final concentration) and 30 mM nitric acid (HNO_3_). The solution is subsequently incubated between 50 and 60°C for 24 h, generating a milky white CPP crystal principate. Monoclinic‐CPP crystals observed in clinical pseudogout have a typical rhomboid shape and are shorter than 10 μm in length. The methods described above have been confirmed to yield crystals between 2 and 15 μm in length.^67^

Crystal preparations can be harvested using filtration apparatus with Grade 5 Whatman Paper (Thomas Scientific, Cat# 4712R25) under negative pressure. Subsequently, they should be washed with either 100% ethanol or methanol and allowed to dry. Importantly, to minimize the risk of microbial contamination, the preparation should be evaluated for endotoxins using an endotoxin assay kit (Genscript, Cat# L00488‐20) to ensure that a sterile inflammatory response is induced. While a negative result does not exclude all forms of contamination, this helps ensure that the observed inflammatory response can be attributed to crystal mediated inflammation.^56^, ^68^, ^69^ In some studies, MSU crystals were additionally autoclaved at 180°C for 2 h to further eliminate endotoxins or dried under UV light.^18^, ^22^, ^33^, ^69^, ^70^ The effects of autoclaving and UV light irradiation on CPP crystal integrity have not been reported. The identification of MSU crystals is confirmed by their needle‐like shape and strong negative birefringence under compensated polarized light microscopy.^71^ In comparison, CPP crystals have a rhomboid shape and exhibit no or weak positive birefringence. The dimensions of the crystals should be confirmed using scanning electron microscopy (SEM).^65^ Briefly, crystal samples can be prepared for SEM analysis by evenly distributing the crystals over conductive tape followed by mounting onto an SEM sample stub. The sample is then coated with a thin layer of gold for 2 min using a sputter coater to enhance resolution. Crystals should be examined under high‐vacuum mode at 15 kilovolts (kV), with acquisition of images from 10 random fields of view at ×1000 magnification. In each field, 10 crystals can be measured and their respective length quantified using SEM inbuilt software.^56^ It is recommended that researchers refine the cited techniques within their laboratory to optimally produce crystals that closely resemble the characteristics of those observed in clinical gout and pseudogout for the reasons mentioned previously. The crystals should be transferred to a 50 mL conical tube and weighed; the weight of the empty tube is subtracted to determine the yield. In preparation experimentation, the desired mass of crystals used to stimulate each air pouch is transferred to induvial 15 mL conical tubes under sterile conditions.

#### Recommendation

Refine and optimize the MSU crystal synthesis methods described by McCarty et al. to yield needle shaped crystals 6–10 μm in length. Use the CPP crystal synthesis methods described by Martinon et al. to yield rhomboid shaped crystals <10 μm in length. Crystal preparations should be confirmed under polarized light microscopy and length quantified using scanning electron microscopy. Crystal preparations should be tested for endotoxins using endotoxin assay kit prior to experimentation.

### 3.2 Ethics

All protocols using live animals must first be reviewed and approved by an Institutional Animal Care and Use Committee (IACUC) or must conform to governmental regulations regarding the care and use of laboratory animals. The generation, induction of inflammation and sampling of the subcutaneous air pouch model is well tolerated, with minimal discomfort arising primarily from needle insertion during pouch formation and fluid collection. To further minimize animal distress, isoflurane anesthesia should be administered during pouch generation, inflammation induction and prior to sampling. Between protocol steps, animals remain conscious, mobile, and pain‐free during the development of the air pouch and following the induction of crystal‐induced inflammation. Moreover, the inflammatory response remains localized within the air pouch, with no evidence of a systemic inflammatory reaction. At the conclusion of the protocol, euthanasia should be performed in accordance with ethical guidelines typically through cervical dislocation, asphyxiation or terminal exsanguination following deep isoflurane anesthesia. Animals should be regularly monitored for signs of distress throughout the protocol. If an animal experiences significant distress, the protocol should be terminated early, and the animal should be humanely euthanised as described above. Compared to other models of gout and pseudogout where crystals are injected into or naturally accumulate in load‐bearing joints, the skin pouch model represents a less painful, lower‐burden alternative by avoiding unnecessary degeneration and inflammation of weight‐bearing joints, which can be severely painful and distressing for animals.^26^ Furthermore, the larger sample volume and repeated sampling afforded by the air pouch enables analysis of a greater number of inflammatory mediators, reducing the number of animals required. These advantages support ethical principles for animal research by contributing to both reduction and refinement in the ethical, humane and responsible use of animals for scientific purposes.

### 3.3 Animals

As shown in Table 4, mice (C57BL/6, CD1 or BALB/c) and rats (Wistar or Sprague Dawley) have been used when applying the model. Murine models are advantageous as the animals are small, readily available, and inexpensive compared to other animals and allow for more reproducible results as experiments can be repeated more frequently to obtain greater confidence in results. Moreover, the molecular mechanisms underlying MSU and CPP crystal inflammation are conserved between rodents and humans.^25^ Based on the review, most studies utilized mice (56 of 83), with the majority being C57BL/6 strain (42 of 56) and aged between 7 and 9 weeks (34 of 56). Most studies using rats predominantly utilized the Sprague Dawley strain (17 of 27), with the age at which animals were investigated rarely being specified. In one study, Wistar rats aged between 10 and 12 weeks were utilized,^56^ while two studies utilized Wistar rats at 7 weeks age.^60^, ^72^ To date, no studies have compared differences in inflammatory responses to MSU and CPP crystals across different mouse and rat strains, making it difficult determine which strain most closely resembles the human inflammatory response. Comparisons between existing studies are further complicated by differences in experimental methodologies, which will be discussed later. Given these limitations, the use of C57BL/6 mice is recommended for future investigations to maintain consistency with existing literature and due to their well‐established translational relevance as a standard model in preclinical research. Moreover, predominately only male animals (82 of 83) have been utilized, with only one study accounting for both sexes.^73^ This greatly limits the generalisability of findings in females, highlighting an important area for future improvement. In accordance with standard animal care guidelines, animals should be acclimated for a week prior to experimentation, housed in groups of at least three under constant temperature, a 12‐h light–dark cycle and with free access to food and water. Performing simultaneous pouch aspirations and treatment with therapeutic agents on groups of up to 8 animals is routine for experienced groups, with new users encouraged to limit pilot studies to 3–4 animal cohorts initially, increasing thereafter. All research groups are recommended to include up to 5% of animals or a minimum of 10 animals for technique refinement within their animal ethics/institutional review board applications, with reproducible pouch inflation proving to be the area requiring most refinement. It is recommended that two researchers be assigned to the protocol, particularly for data collection. One researcher should be responsible for handling animals and collecting pouch fluid, while the other should perform sample preparation, including analysis such as total and differential cell counting, which will be discussed later. This allows for efficient coordination, as sample collections are conducted on groups of animals at each time point. Implementing these standardized practices will enhance experimental efficiency and ensure the reproducibility of findings.

**TABLE 4:** Parameters for experimental animals.

Variable	Recommended (published ranges)
Age	Mouse: 7–9 weeks (6–16 weeks) Rat: 10–12 weeks (7–12 weeks)
Sex	Female and male (male)
Strain	Mouse: C57BL/6 (CD1, BALB/c) Rat: Sprague Dawley (Wistar)
Diet	Standard chow
Acclimatization duration	1–2 weeks
Light–Dark cycle	12 h

#### Recommendation

Utilize male and female C57BL/6 mice aged 7–9 weeks, acclimated for 1 week prior to experimentation, housed in groups of three, under constant room temperature and a 12‐h light–dark cycle, with free access to food and water and standard chow diet.

### 3.4 Generation of the subcutaneous air pouch

**FIGURE 2:**
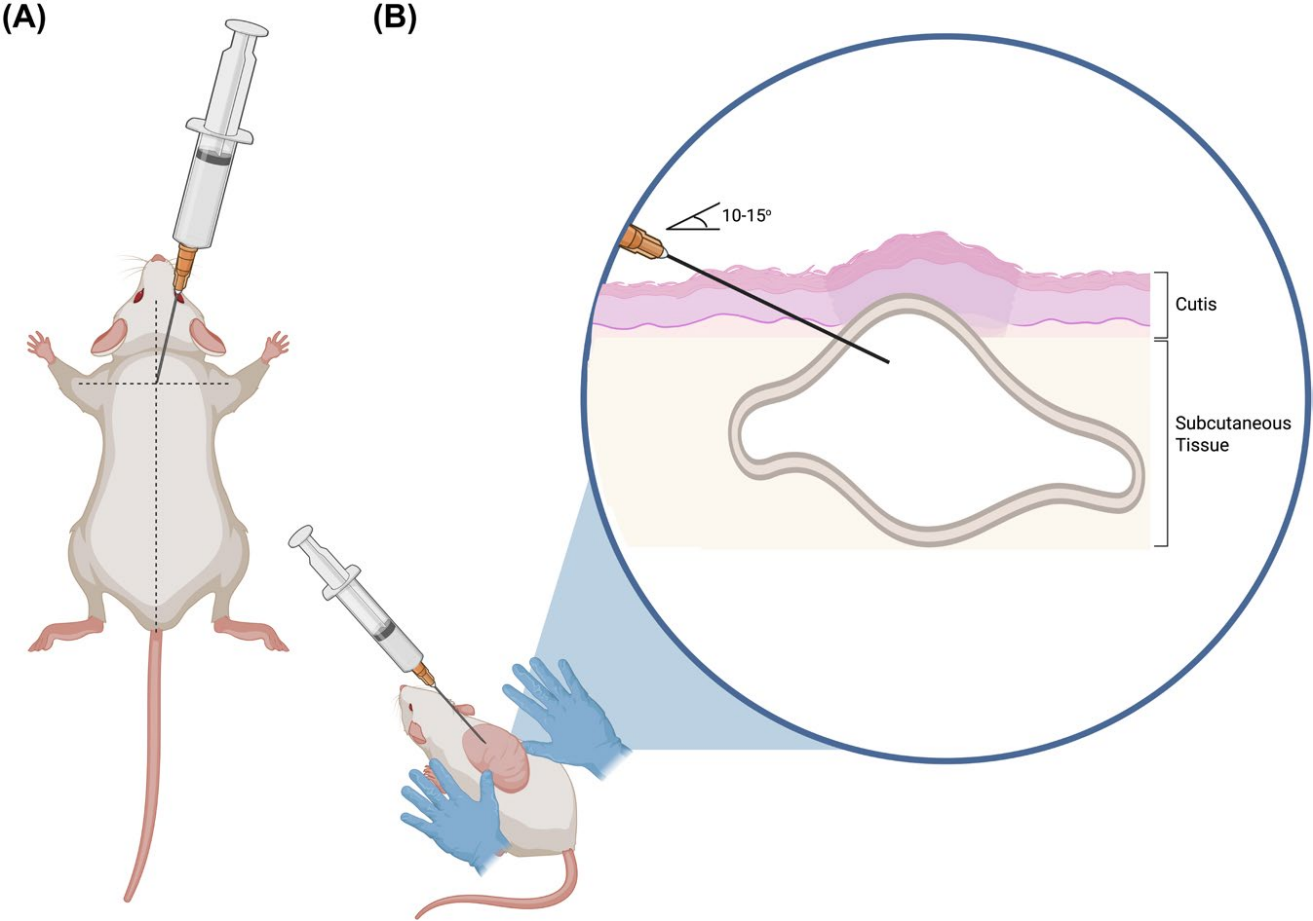
Generation of the air pouch. (A) The skin between the scapula on the dorsal surface is located. (B) Sterile air is injected into the subcutaneous space to generate the pseudo‐synovial joint by the primary researcher. The shape of the pouch is molded on each side using a second researcher's fingers as it is inflated. Figure created using biorender.com.

The method of generating the subcutaneous air pouch is not technically challenging and takes <5 min per animal. Table 5 summarizes the range of parameters for generation of the subcutaneous air pouch. Animals should be anesthetized (0%–4% inhalation isoflurane, 1 L/min 100% O_2_) prior to and during the procedure and placed prone to access the dorsal surface. Fur should be removed from the area (approximately 3 × 3 cm for rats and 1.5 × 1.5 cm for mice) over which the pouch will be inflated with electronic hair trimmers close to the skin surface and sterilized with liberal 70% v/v ethanol. As shown in Figure 2A,B, with the rodent's snout facing the primary researcher, the skin between the scapulae should be raised, forming a triangular shape. Room air should then be drawn into a syringe and delivered by the primary researcher into the subcutaneous space between the dermis and the subcutaneous tissue of the dorsal surface of the rodent between the scapulae via a 27 G needle with a 0.2‐μm sterile filter attached between the syringe and the needle to ensure delivery of sterile air. Studies have reported delivery of 20 mL (10–30 mL) in the rat and 5 mL (2–5 mL) in the mouse. The needle should be inserted at a shallow angle (10°–15°) and approximately 5 mm into the skin within the invagination where reduced resistance is encountered, with air transferred slowly and consistently into the subcutaneous space, ensuring air is not injected into the muscle. A small test injection can confirm correct placement, as successful entry into the subcutaneous space results in the formation of a small bleb. It is important to place the second researcher's fingers laterally, using gentle pressure, on each side of the area where the pouch is to be inflated, to ensure formation of a single cavity and prevent the air pouch forming along the animal's flank. Syringe needles with larger diameters may create a larger wound site, increasing the risk of the pouch deflating due to inadequate sealing. Upon needle removal, the puncture site should be compressed (pinched) lightly for 20 s to generate a seal. To maintain patency of the air pouch, most studies (79 of 83) delivered a smaller additional volume of air using the same method on the 4th day (Day 3).^22^, ^55^, ^74^ Studies report delivery of 10 mL (10–22 mL) in the rat and 3 mL (1.5–5 mL) in the mouse. In a few studies (15 of 83), prior to stimulation of inflammation, further additional volumes (rat: 10–15 mL; mouse: 3 mL) of air were delivered multiple times over time due to inadequate pouch inflation^61^, ^75^, ^76^, ^77^, ^78^, ^79^, ^80^ There was a lack of consistency in some studies regarding the amount of air delivered or whether additional volumes of air were administered to individual animals. The impact of varying air volumes between inflations, as well as additional air inflations of the pouch, on the subsequent inflammatory response remains unclear. Based on experience, the authors recommend maintaining consistency in the amount of air delivered between animals to ensure standardization and reproducibility of findings. After each inflation, animals should be monitored until they are fully conscious and moving.

**TABLE 5:** Parameters for generation of the subcutaneous air pouch.

Variable	Recommended (published range)
Needle	27 G
Air filter	0.2 μm
First day inflation volume (Day 0)	Rat: 20 mL (10–30 mL) Mouse: 5 mL (2–5 mL)
Fourth day second inflation volume (Day 3)	Rat: 10 mL (10–22 mL) Mouse: 3 mL (1.5–5 mL)
Additional inflations given every 2–3 days prior to stimulation of inflammation	Rat: 10 mL (10–15 mL) Mouse: 3 mL (3 mL)

#### Recommendation

Delivery of sterile room air through a 27G needle and a 0.2 μm filter on Day 0 (rat: 20 mL; mouse 5 mL) and Day 3 (rat 10 mL; mouse 3 mL), using the technique described above.

### 3.5 Induction of sterile crystal inflammation

The duration of pouch inflation or the age of the pouch prior to stimulation with crystals likely determines the intensity of the inflammatory response. In studies investigating MSU crystal inflammation, stimulation of pouches that have been inflated for longer periods generated a more intense inflammatory response, characterized by increased leukocyte infiltration.^24^ Table 6 summarizes the range of parameters for stimulation of the subcutaneous air pouch. The pouch is most often (43 of 83) stimulated on the 7th day (Day 6). This longer time frame likely allows the fibrocyte‐like cells to proliferate more extensively and develop a more mature membrane lining that can trigger a stronger inflammatory response.

**TABLE 6:** Parameters for stimulation of the air pouch with MSU and CPP crystals.

Variable	Recommended (published range)
Needle	21 G
Age of pouch at the time of crystal stimulation	Rat: Day 6 (Day 1–8.5) Mouse: Day 6 (Day 2–7)
Amount of crystals	MSU Rat: 15 mg (5–150 mg) Mouse: 3 mg (0.5–20 mg) CPP Rat: 30 mg (5–150 mg) Mouse: 1 mg (1 mg)
Suspension solution	MSU Rat: Saline (HEPES BSS, PBS, Saline) Mouse: PBS (HBSS, PBS, Saline) CPP Rat: Saline (HEPES, PBS, Saline) Mouse: PBS (Stain Buffer)
Suspension volume	MSU Rat: 5 mL (2–15 mL) Mouse: 1 mL (0.3–5 mL) CPP Rat: 10 mL (5–15 mL) Mouse: 0.5 (0.5 mL)
Pouch massage time	1 min

Abbreviations: HBSS, Hanks' balanced salt solution; HEPES BSS, 4‐(2‐hydroxyethyl)‐1‐piperazineethanesulfonic acid buffered saline solution.

The crystal preparation should be suspended in sterile saline or phosphate buffered saline (PBS), sonicated for 10–20 min to disperse the crystals, and then drawn into a syringe. The use of 3 mg MSU crystals for every 1 mL of suspension volume was the most common (27 of 56) suspension composition reported for the mouse. There was considerable variation in suspension composition for rats; however, the most common (7 of 27) was 2 mg of MSU crystals per 1 mL of suspension volume. Moreover, there is insufficient evidence to guide the choice between PBS and saline for suspension solution. Most studies in the mouse used PBS (44 of 56), while most studies used saline in the rat (21 of 27). The impact of each solution on the pouch environment and the resulting inflammatory response has not yet been elucidated. Phosphate‐buffered saline may offer greater stability for MSU crystals due to its pH buffering capacity. Based on our review, a standardized composition of 3 mg of MSU crystals per 1 mL of suspension volume is recommended for consistency with the existing literature. Specifically, 15 mg in 5 mL saline for rats and 3 mg in 1 mL PBS for mice are suggested. A greater number of crystals and larger suspension volumes are advised for rats due to the larger size of the air pouch. The intensity of the inflammatory response is likely proportional to the number of crystals and the volume of suspension solution introduced into the pouch. In a study investigating MSU and CPP crystal inflammation, delivery of the same amount of crystals with larger volumes of suspension solution (10 mL vs. 1 mL) likely facilitates greater dispersion of the crystals throughout the cavity and therefore leads to a stronger inflammatory response.^24^ There are too few studies to determine the optimal CPP crystal suspension composition for the model in mice (2 studies) and the rat (6 studies). In mice, one study suspended 1 mg of crystals in 0.5 mL of stain buffer, while the other used 1 mg of crystals per 1 mL PBS. In the rat, 32.5 mg (5–150 mg) CPP crystals in 10 mL (5–15 mL) suspension volume was used. There was significant variation is crystal suspension compositions between studies. Saline was most often used (3 of 6), and may be preferred over PBS as it would result in reduced phosphate interactions that could potentially alter the structural integrity of CPP crystals. Based on these limited studies, a suspension composition of 1 mg CPP crystals in 0.5 mL PBS for mice and 30 mg CPP crystals in 10 mL saline for rats is recommended, as these formulations best align with the existing literature.

Under sedation with the animal either prone or on their side, the suspension is delivered into the pouch through a syringe via a 21G needle, from the same direction as the air inflation. Difficulty in delivering the suspension is encountered when using smaller diameter needles due to aggregation of the crystals in solution. Thus, it is important to keep the crystals in a homogenous suspension by agitation of the solution directly before injection. After delivery of the crystal suspension, the insertion point is pinched as the needle is removed to minimize pouch deflation. The pouch is gently massaged for 1 min to facilitate dispersion of the crystals throughout the cavity. The procedure may cause the air pouch to deflate; however, this is likely not significant as the membrane lining has already formed. Despite this, aspirating fluid from the pouch can prove more challenging if pouch deflation occurs.

#### Recommendation

On Day 6, deliver MSU crystals (rat: 15 mg in 5 mL saline; mouse: 3 mg in 1 mL PBS) and or crystals (rat: 30 mg, 10 mL saline; mouse: 1 mg, 0.5 mL PBS) via a 21G needle followed by a 1 min massage of the pouch using the methods described above.

### 3.6 Harvesting pouch fluid

The inflammatory response elicited by MSU and CPP crystals induces pronounced accumulation of inflammatory exudate, henceforth termed ‘pouch fluid’, within the pouch cavity, which can be analyzed for cell infiltration and inflammatory mediators. Pouch fluid can be harvested using several techniques as summarized in Tables 7 and 8. The evolution of the inflammatory response over time can be evaluated by repeatedly aspirating the pouch cavity with a 21G needle connected to a syringe, using the same approach as for air inflation and crystal administration.^36^ For the purpose of time course studies, performing multiple collections on the same animal allows for reduced numbers of animals to be used. Repeated sample collections have been performed for up to 48 and 178 h at variable intervals after MSU and CPU administration, respectively. Collections were most often performed at 6 (60 of 83), 12 (21 of 83) and 24 (38 of 83) h post crystal delivery. It is conceivable that repeated sampling could attenuate the severity and progression of the inflammatory event by removal of inflammatory exudate and crystals. This effect may be particularly relevant in studies where the endpoint involves assessing treatment efficacy during the peak or sustained phase of inflammation, as observed in severe inflammatory attacks. This can be mitigated through the inclusion of appropriate control groups and alignment of sampling frequency with the study's specific endpoints.

**TABLE 7:** Parameters for direct aspiration or infusion and withdrawal method for exudate collection.

Variable	Recommended (published range)
Needle	21G
Infusion solution (not applicable for direct aspiration method)	Rat: saline (saline, PBS, PBS or saline with 2.5–10 mM EDTA) Mouse: PBS (saline, PBS, stain buffer, PBS or saline with 2.5–10 mM EDTA, PBS or saline with 20 units/mL heparin)
Infusion solution volume (not applicable for direct aspiration method)	Rat: 5 mL (5 mL) Mouse: 2 mL (2–5 mL)
Technique	Rat: 5 mL delivered and 5 mL withdrawn (5 mL of delivered and 4 mL withdrawn, pouch fluid directly aspirated without delivery of PBS/saline) Mouse: 2 mL delivered, and 2 mL withdrawn (2 mL delivered and 1.5 mL withdrawn, pouch fluid directly aspirated without delivery of PBS/saline)
Time points (post crystal delivery)	6, 12, 24, 48 h (1–168 h)

**TABLE 8:** Parameters for dissection method for exudate collection.

Variable	Published range
Washing solution	PBS Saline PBS or saline with EDTA (2.5–10 mM) PBS or saline with heparin (20 units/mL)
Wash solution volume	Rat: 5 mL Mouse: 1–2 mL
Pouch fluid retrieval method	Fixed volume of solution used to wash pouch, then aspirated Pouch fluid directly aspirated without washing
Time points (post MSU delivery)	6, 12, 24, 48 h (1–168 h)

Under brief anesthesia, the animal is placed on their side to allow the exudate to collect on the side of the pouch that is facing down, maximizing the volume of exudate that can be collected. Either cranially or caudally, the entry point of the needle should be approximately where the exudate accumulates. The pouch is accessed via different sites in order not to disturb the healing site and minimize trauma caused by repeated needle insertions at the same location. This method of *direct pouch aspiration* was utilized in 5 studies. The volume or mass of pouch fluid retrieved can be quantified as this correlates with the strength of the inflammatory response.^24^, ^48^ The time points at which the pouch fluid is harvested correspond to different phases of the inflammatory response. Leukocyte infiltration after MSU administration occurs from 8 h (6–9 h) and 6 h (6–10.5 h) in the mouse and rat, respectively. In studies investigating CPP crystal inflammation in the rat, leukocyte infiltration typically occurs between 6 and 12 h following crystal administration. Due to the limited number of studies, it is difficult to ascertain the evolution of the leukocyte response to CPP crystals in mice. One study reported a higher leukocyte concentration at 6 h compared to 24 h,^7^ suggesting a similar response to the rat. Importantly, the collection time points are chosen so as to adequately capture the rise, peak and resolution in concentration of the analytes of interest, which is discussed in Section 3.10. Table S1 in supporting information summarizes the time points reported in the literature at which leukocyte infiltration and various analytes were assessed. However, differences in sampling intervals between studies make it difficult to establish when exactly peak leukocyte infiltration or cytokine accumulation occurs. This conclusion is also likely confounded by differences in methods for creating the model. Therefore, time course experiments are utilized to verify the points of peak concentration, prior to testing potential therapeutic strategies. Based on our review, preliminary studies should capture leukocyte counts at 6, 12, and 24 h. We recommend an additional collection at 48 h to assess inflammation during the resolution phase of the inflammatory response. In the authors' experience, caution is advised when performing a 1‐h collection as a significant volume of crystals are retrieved from the pouch. The number of time points utilized should be chosen based on the study aims, as the amount of exudate within the pouch reduces overtime, making consecutive collections difficult to perform. Particularly during initial collections, resistance can be encountered when recovering pouch fluid due to crystal aggregations. To overcome this, whilst massaging the pouch, small volumes can be gently reinfused and withdrawn until the desired volume is collected. Repeated aspirations may cause trauma and blood contamination of the exudate, which can confound results due to the presence of heme and introduction of circulating leukocytes. The presence of blood can be minimized by aspirating the pouch at different locations between time points. If the exudate is proving difficult to retrieve (e.g. there is considerable resistance during aspiration), the collection should be voided to avoid damage to the tissue environment caused by repeated collection attempts.

Alternatively, the method can be modified by *delivering and withdrawing* a fixed volume of saline or PBS (e.g. rat: 5 mL; mouse: 2 mL) at each collection to allow for easier pouch aspiration.^28^ This method was utilized by 31 of 83 studies. For this method, the pouch is gently massaged for 1 min prior to each aspiration to ensure the exudate composition is homogenous. There is insufficient evidence to guide whether it is better to inject saline or PBS into the pouch to retrieve pouch fluid in the model. However, across studies, saline was more often used in the rat (5 of 8) and PBS in the mouse (20 of 23). To further refine the technique above, a fixed volume of the injected saline or PBS can be left in the pouch to prevent a traumatic collection caused by attempting to recover the entire volume. In the rat, 5 mL of saline can be delivered into the pouch and 4 mL withdrawn at each time point leaving 1 mL of residual saline.^53^, ^56^ In the mouse, 2 mL of PBS can be delivered into the pouch and 1.5 mL withdrawn, leaving 0.5 mL residual PBS in the pouch. However, with this method, the volume of exudate produced cannot be used to quantify inflammation, as an additional volume of solution is being introduced into the pouch with each collection. Moreover, this approach dilutes the pouch cavity with each collection, therefore, inclusion of a control group (i.e. no therapeutic treatment) is required for comparison. To overcome this, results could also be expressed relative to fluid protein content, as this would correspond to the degree of inflammation; however, this approach has not been widely evaluated. In other studies, an indwelling catheter has been placed into the pouch for the entire duration of the study, allowing continuous access to the pouch cavity.^61^, ^64^, ^81^ In some studies, ethylenediaminetetraacetic acid (EDTA) or heparin have been incorporated into the infusion solution to inhibit cell aggregation within the collected pouch fluid.^37^, ^47^, ^48^, ^50^, ^52^, ^82^

Alternatively, animals can be euthanised at each time point and the pouch cavity can be accessed using blunt dissection along a sagittal plane, which is described in Section 3.7. The pouch fluid can then be directly aspirated using a needle/syringe or transfer pipette (14 of 83 studies).^59^, ^78^, ^79^, ^83^ In some studies, the cavity was washed using a fixed volume of saline or PBS administered via syringe or transfer pipette prior to aspiration (12 of 83 studies).^51^ Although these methods would likely improve reproducibility of data obtained, they would result in substantially greater numbers of animals required to perform the experiment, as animals would need to be euthanised after each time point. Therefore, a preliminary time course study is completed initially to establish time points relevant for the study aims.

#### Recommendation

As described above, injection of a fixed volume saline or PBS (rat 5 mL saline; mouse 2 mL PBS), followed by a 1 min massage of the cavity and subsequent withdrawal of an equivalent volume of pouch fluid (rat 5 mL; mouse 2 mL) via a 21G needle at 6, 12, 24, and 48 h post induction of inflammation.

### 3.7 Pouch fluid cell count

In most studies that did not utilize EDTA/heparin to infuse or wash the pouch, the exudate was later combined with EDTA (5 mM) or heparin (20 units/mL) to inhibit cell aggregation.^30^, ^37^, ^47^, ^48^, ^50^, ^52^, ^58^, ^61^, ^78^, ^82^, ^84^, ^85^, ^86^, ^87^ This method may be preferable as it minimizes interference with the native pouch environment. The pouch fluid should be kept cold (4°C, on ice) after collection to minimize degradation of inflammatory mediators. The cells should be pelleted via centrifugation (1000× *g*, 8 min, 4°C) and the supernatant aliquoted for subsequent analysis of inflammatory mediators. If analysis of inflammatory mediators cannot be performed immediately, the supernatant should be promptly flash frozen using liquid nitrogen and stored at −80°C. The cell pellet should be resuspended in 0.5 mL ice‐cold PBS (volume kept consistent). A total leukocyte count can be easily obtained using a hemocytometer, automated cell counter,^40^ or using flow cytometry with an anti‐Cluster of Differentiation (CD) 45 antibody. A differential cell count, to determine the proportion of subpopulations of leukocytes, can be easily performed manually using a rapid differential staining kit (Diff‐Quick or Wright Stain),^52^, ^53^, ^56^, ^69^ or using flow cytometry with conjugated monoclonal antibodies.^7^, ^29^, ^39^, ^41^, ^48^, ^49^, ^68^, ^75^, ^78^, ^79^, ^80^, ^82^, ^88^, ^89^, ^90^ A key limitation in the literature is inconsistency in measuring leukocyte counts, with studies reporting different units such as total cells per pouch, cells per milliliter of exudate, or extravasation rate per hour. This significantly limits comparison and synthesis of findings across studies. Our recommendation is to quantify leukocytes using cells per milliliter of exudate (cells/mL) as it reduces variability due to differences in fluid volume, ensuring data is reliable regardless of exudate recovery.

#### Recommendation

Collect pouch fluid in 5 mM EDTA additive followed by centrifugation (1000× *g*, 8 min, 4°C). Immediately analyze the pellet to obtain total and differential cell counts using a hemocytometer and Diff‐Quick stain, respectively. Total cell count should be measured as cells per milliliter of exudate. Immediately store the supernatant at −80°c for later analysis.

### 3.8 Pouch tissue analysis

If repeatedly aspirating the pouch, at the final collection, the animals are euthanised and desired tissue samples can be collected to complement exudate analysis. If pouch fluid is collected by euthanising the animal and dissecting the pouch at desired time points, tissue samples can be simultaneously collected. The histopathology of the pouch tissue can be visualized using standard Hematoxylin & Eosin (H&E) staining. The apex of the pouch membrane is exposed by making a subcutaneous T‐shaped incision into the dorsal skin overlying the pouch with scissors or a scalpel. The membrane can be separated from the overlying skin and adjacent subcutaneous and paraspinal tissue by blunt dissection. The pouch membrane can then be grasped with forceps, elevated, and cut at the base with scissors. Care must be taken to avoid removal of paraspinal and nuchal tissues, which typically adhere to the base of the membrane. The isolated membrane can be post‐fixed in 10% paraformaldehyde or 70% ethanol.^24^, ^32^, ^33^, ^34^, ^38^, ^42^, ^47^, ^55^, ^64^, ^78^, ^79^, ^83^, ^85^, ^86^ As shown in Table 9, inflammation within the pouch tissue can be quantitatively assessed using H&E‐stained 5 μm tissue sections. A blinded investigator scores the degree of inflammatory cell infiltration and measuring membrane thickness, as these correspond to the severity of inflammation.^41^ A scale ranging between 0 and 4 is used, with 0 representing the lowest and 4 representing the highest value observed. The severity of inflammation can be graded based on the sum of individual scores for polymorphonuclear and mononuclear cell infiltration. Pouch tissue can also be evaluated for changes in inflammatory protein and gene expression, particularly IL‐1β, TNF‐α and IL‐6 as they play an integral role in the progression of inflammation in gout and pseudogout.^35^, ^70^, ^74^, ^91^ The pouch tissue of the animals used for technique refinement can be utilized to optimize staining protocols.

**TABLE 9:** Histological scoring system for quantifying inflammation in the air pouch model.^41^

	Cell types	Score				
		0	1	2	3	4
Inflammation	Polymorphonuclear cells (neutrophils and eosinophils)	None	Minimal, 1–5/per HPF	Slight, 6–15/per HPF	Moderate, 16–25/per HPF	Severe, >25/per HPF
	Mononuclear cells (lymphocytes and macrophages)	None	Minimal, 1–5/per HPF	Slight, 6–15/per HPF	Moderate, 16–25/per HPF	Severe, >25/per HPF
Membrane thickness of murine air pouch		None	<50 μm	50–75 μm	76–100 μm	>100 μm

*Note*: The severity of inflammation is graded based on the sum of individual scores for polymorphonuclear and mononuclear cell infiltration.

Abbreviations: HPF, high power field (40×); μm, micrometer.

#### Recommendation

Dissection of the pouch tissue after euthanasia using the technique described above. Tissue fixation in 10% paraformaldehyde, followed by tissue processing and H&E staining according to local guidelines, with prior optimisation. A researcher blinded to the experimental design should assess and grade inflammation within the pouch tissue using the system described in Table 9.

### 3.9 Treatment with potential anti‐inflammatory agents

Investigation of potential treatments for gout and pseudogout commonly occurs both prior to (pre‐treatment) or after stimulation of inflammation depending on whether the study is validating a novel target or assessing the clinical utility of a new therapeutic. Regarding pre‐treatment, the desired therapeutic agent can be administered such that its serum concentration peaks just after stimulation of the pouch with crystals.^14^, ^53^, ^56^ Therefore, the pharmacokinetics of the therapeutic agents can be investigated with the intended corresponding route of administration, to optimize timing of their administration. Alternatively, therapeutics can be administered periodically each day after induction of inflammation to replicate how gout and pseudogout would be treated clinically.^82^ If the pharmacokinetic properties of the potential treatment are unknown, the therapeutic agent can be delivered directly into the pouch via a 25G needle.^27^, ^34^, ^39^, ^58^, ^63^, ^80^, ^90^

#### Recommendation

Pre‐deliver therapeutic agents with their peak serum concentration coinciding with crystal induction.

### 3.10 Animal groups

Our review revealed mean sample sizes of 6 (3–19) and 5 (3–16) per group in the rat and mouse, respectively. The authors recommend that a minimum of six animals should be allocated per group, due to inherent variability in the method. Treatment groups are compared to a vehicle group (MSU/CPP suspension delivered into pouch with vehicle for treatment). A sham group (solution without MSU/CPP delivered into pouch with saline for treatment) should also be included to confirm that the inflammatory response observed is the result of crystal‐induced inflammation and not infection/procedural approach. A therapeutic positive control group (MSU/CPP suspension delivered into pouch with established anti‐inflammatory for treatment) should also be included so that experimental drugs can be compared to conventional therapies. For example, treatment with steroidal anti‐inflammatory drugs dexamethasone and prednisolone, and the non‐steroidal anti‐inflammatory drugs carprofen and indomethacin significantly downregulate inflammation in the model.^31^, ^53^, ^55^, ^69^, ^74^, ^82^, ^88^ Pre‐treatment with prednisolone prior to crystal administration may be preferred as a standard comparator, given its wide use in the acute management of crystal arthropathies.^92^ Table 10 summarizes the treatment protocols and administration parameters used in the subcutaneous air pouch model to study MSU and CPP crystal inflammation.

**TABLE 10:** Parameters for experimental groups.

	Positive control	Treatment(s)	Vehicle	Sham
Pouch stimulation	Suspended MSU/CPP crystals into pouch	Suspended MSU/CPP crystals into pouch	Suspended MSU/CPP crystals into pouch	Same volume of suspension solution (without MSU/CPP crystals) into pouch
Treatment^a^	Dexamethasone Pre‐treatment option i.p. 2 mg/kg 2 h prior to MSU delivery^31^ i.p. 20 mg/kg daily for 3 days prior to crystal delivery^82^ Post‐treatment option i.p. 6 mg/kg 24 h post crystal delivery^74^ Prednisolone Pre‐treatment option i.p. 10 mg/kg 30 min prior to crystal delivery^88^ Post‐treatment option i.p. 20 mg/kg 24 h post crystal delivery^74^ Carprofen Pre‐treatment option i.p. 5 mg/kg daily for 3 days prior to crystal delivery^53^ Indomethacin Pre‐treatment option i.v. 2 mg/kg immediately prior to^59^ i.p. 50 mg/kg immediately prior to^59^ i.p.0.5 mg/kg daily for 4 days prior to crystal delivery^69^ p.o.20 mg/kg 30 min prior to crystal delivery^55^ Colchicine Pre‐treatment option s.c. 0.5 mg/kg 2 h prior to crystal delivery^93^	Treatment compound dissolved and delivered via desired administration route	Volume of solvent via same administration route for treatment	Volume of solvent via same administration route for treatment
Treatment Solvent	DMSO diluted with saline	DMSO diluted with saline	DMSO diluted with saline	DMSO diluted with saline

Abbreviations: DMSO, dimethyl sulfoxide; i.p., intraperitoneal; i.v., intravenous; p.o., per oral; s.c, subcutaneous.

^a^ Ideally the same solvent, solvent volume and administration route are utilized across all groups.

#### Recommendation

Utilize groups of at least 6 animals and include a vehicle group, sham group and positive control group receiving prednisolone 5 mg/kg i.p. 30 min prior to crystal delivery.

### 3.11 Analysis of inflammatory mediators

The pouch fluid is consistently analyzed in published studies to assess changes in cytokine and eicosanoid release and components of the oxidative stress response.^30^, ^85^, ^88^ These compounds can be measured using commercially available enzyme‐linked immunosorbent assay (ELISA), multiplex bead‐assay and biochemistry assays, western blot or polymerase chain reaction (PCR). Time points for analysis are based on the research question and when analytes are expected to be physiologically relevant or above the limit of detection of an assay. The impact of treatment agents on the morphology of the inflammatory response should also be taken into consideration. This emphasizes the importance of a preliminary time course study to determine optimal time points for leukocyte and inflammatory cytokine assessment. As mentioned, Tables S1 and S2 in supporting information detail the peak time points for various hematological and biochemical markers reported in the literature for MSU and CPP crystals, respectively. The cytokines most often measured were IL‐1β (42 of 83), IL‐6 (21 of 83), TNF‐α (19 of 83) and CXCL‐1 (15 of 83). Overall, the authors recommend analysis of total leukocyte infiltration, cytokines IL‐1β, IL‐6 and TNF‐α and quantitative pouch tissue analysis as a baseline in this model.^94^ The aforementioned inflammatory cytokines were chosen given their established role in inflammatory arthropathies and clinical relevance as therapeutic targets for gout and pseudogout. However, there was considerable variability in analyte concentrations across studies, even at corresponding time points, limiting direct comparisons between studies. This is likely explained by methodological differences, including the age of the air pouch, the number of crystals delivered, the frequency of sample collection, and dilution effects depending on whether a solution was injected into the pouch. Tables 11 and 12 summarize the median peak concentration time points and observed ranges for relevant cytokines across studies on MSU and CPP crystal‐induced inflammation, respectively.

**TABLE 11:** Time points for anticipated peak leukocyte and relevant cytokine concentrations for MSU crystal inflammation.

	Peak median (IQR)	
	Mouse	Rat
Total leukocyte count	8 h (6–9 h)	6 h (6–10.5 h)
IL‐1β	6 h (3.5–7 h)	Insufficient data
IL‐6	6 h (3–6 h)	Insufficient data
TNF‐α	6 h (4.75–6.5 h)	Insufficient data

**TABLE 12:** Time points for anticipated peak leukocyte and relevant cytokine concentrations for CPP crystal inflammation.

	Peak median (range)	
	Mouse	Rat
Total leukocyte count	Insufficient data (6 h) ^7^, ^68^	6 h (6–12 h) ^24^, ^60^, ^61^, ^72^
IL‐1β	Insufficient data (6 h) ^7^	Insufficient data
IL‐6	Insufficient data	Insufficient data
TNF‐α	Insufficient data	Insufficient data

#### Recommendation

Analysis of at least IL‐1β, IL‐6, and TNF‐α using standard ELISA kits. Conduct preliminary study to identify peak analyte concentration time points, followed by targeted analysis of analytes at their respective peaks time points.

## 4 LIMITATIONS AND FUTURE RESEARCH

Future studies on the air pouch model should focus on standardized methods, with protocol amendments that align with experimental endpoints. All reviewed studies utilized either mice or rats. The preference for murine models is likely attributed to the conservation of key inflammatory pathways between rodents and humans.^25^, ^95^ Despite this, species‐ and strain‐specific variations in immune responses, cytokine production, and inflammatory mediators may impact the reproducibility and translatability of findings in the air pouch model.^49^, ^95^ Future research comparing inflammatory pathways across strains may explain differences in experimental findings, improving their relevance in translational research. Furthermore, most reviewed studies included exclusively male rodents, significantly limiting understanding of potential sex‐based differences in disease pathophysiology and therapeutic responses.^95^, ^96^ Given that hormonal, genetic, and immune differences can influence inflammatory processes, it is essential to expand research to include female animals.^95^, ^97^ Investigating sex‐specific differences in MSU and CPP crystal deposition, inflammatory mediators, and joint damage could provide novel insights into disease pathophysiology and treatment efficacy. Future studies should balance sex representation in models to ensure translational relevance of preclinical research to both male and female patients.

As mentioned previously, variations in the volume of air administered during inflation, number of inflations, age at which the pouch is stimulated with crystals, composition of the crystal suspension, and method of pouch exudate retrieval can influence the inflammatory response.^24^ Larger air volumes create a greater surface area lined with cells that interact with the crystals, while more mature pouches allow additional time for pseudo‐membrane formation and maturation, likely leading to a stronger inflammatory response.^24^ The degree of inflammation is also proportional to the number of crystals delivered. Similarly large volumes of suspension facilitate greater dispersion of crystals within the pouch, leading to a stronger inflammatory response.^24^ Standardizing air pouch generation, including inflation protocols, timing of stimulation, and the composition of the crystal suspension will ensure reproducibility of results across studies. Additional studies assessing inflammatory responses in the model to different crystal preparations, particularly m‐CPP and t‐CPP, may provide valuable insights into crystal arthropathies from initial onset to recurrent flares and long‐term disease evolution.^7^ There is also insufficient evidence to establish whether PBS or saline is better as the crystal suspension and pouch fluid retrieval solution. Phosphate buffered saline may provide greater stability for MSU crystals due to its greater pH buffering capacity. In contrast, saline may minimize phosphate interactions that could alter CPP crystal integrity. Future studies should assess the impact of suspension conditions on crystal stability and experimental outcomes.

There was also significant variation in the methods used to collect pouch fluid across studies, which reflects differences in experimental objectives and research experience. Most studies performed consecutive aspirations of the pouch over time for the purpose of monitoring the evolution of the inflammatory response and responses related to therapeutic delivery.^81^ Although this perturbs the tissue environment, it reduces the number of animals needed to be used. Moreover, the strength of the inflammatory response to MSU and CPP crystals can vary between animals, making repeated sampling within the same animal beneficial for accounting for inter‐animal variability.^18^, ^23^ Direct aspiration of the pouch is mostly employed due to its simplicity and ability to preserve the natural inflammatory environment. It also allows for quantification of the volume of inflammatory exudate produced.^23^ In contrast, the infusion and withdrawal method, involving injection of a fixed volume of saline or PBS before collection, facilitates easier retrieval and reduces variability in collection efficiency.^53^ This approach is particularly useful for studies requiring frequent sampling, as it minimizes resistance during aspiration. However, this dilutes the exudate, which should be accounted for when analyzing pouch fluid composition and highlights the importance of including a vehicle group.^56^ One potential approach is to express results relative to protein concentration, although this has not yet been robustly evaluated, and further studies are needed to validate its reliability. Blunt dissection of the cavity followed by aspiration offers the advantage of high‐quality sample retrieval but is limited to end‐point analyses.^41^ However, it is impractical for time course investigations due to the increased number of animals required. The selection of a collection method should align with study objectives, with time course studies favoring repeated aspiration, while terminal experiments favor pouch dissection for optimal sample yield. Researcher experience also plays a role, as more experienced groups may implement complex protocols with continuous sampling, whereas newer investigators may prefer direct aspiration. Recognizing these methodological differences is important for standardization, as variations in collection techniques can influence the interpretation and comparability of findings. The collection time points and frequency of collections are also guided by the experimental protocol endpoints, and therefore vary greatly between studies.^23^ Different phases of the inflammatory response are captured at various intervals. As demonstrated in Table S1 in supporting information, leukocyte infiltration often peaks at around 8 h in the mouse and 6 h in the rat after MSU crystal administration. Likewise, cytokine concentrations peak at around 6 h, with some studies showing IL‐1β levels preceding TNF‐α and IL‐6 concentrations, likely due to the central role of NLRP3 and IL‐1β in crystal arthritis.^1^, ^9^, ^11^ However, inconsistencies in methods across studies such as the number of crystals injected, age of the pouch, sampling intervals and dilutional effects of injecting fluid into the pouch complicate the determination of peak inflammatory phases and comparison between studies. Studies investigating CPP crystal inflammation in the model are limited. Therefore, preliminary time course experiments are recommended to establish relevant time points before testing potential therapeutic strategies. Based on our review, for time course studies, sampling at 6, 12, 24 and 48 h is recommended for measurement of cell counts as these time points capture the progression of the inflammatory response from onset through peak to resolution. Caution is advised when performing a 1‐h collection as a significant volume of crystals are retrieved from the pouch, which can attenuate the subsequent inflammatory response.^18^, ^56^ Subsequent collection points for analysis of analytes should be determined based on when analytes are expected to peak. Standardization of the above methods will allow for better comparisons between studies.

Each experimental group should consist of at least 6 animals for sufficient power in the study. All future studies should utilize a sham group to account for protocol related effects, a vehicle control group receiving no treatment for comparison with treatment groups, and a positive control group to establish the efficacy of conventional therapies on the model and for comparison.^53^, ^56^ Pre‐treatment with prednisolone prior to crystal administration effectively attenuates the inflammatory response and should be considered as a standard comparator, given its conventional use in the acute management of crystal arthropathies.^92^ Based on the review, candidates for therapy should be ideally administered such that their serum concentration peaks during crystal stimulation, allowing for a direct assessment of their impact on the acute inflammatory response. Appropriate and standardized sample handling is also critical in maintaining data integrity.^23^ Pouch fluid should be combined with EDTA to prevent cell aggregation and kept on ice (4°C) until use. Manual cell counts using a hemocytometer and differential cell counts using differential stain are reliable, cost effective and allow for rapid analysis following pouch fluid collection without the need for additional sample processing and storage, unlike flow cytometry.^18^, ^23^ Based on our review, to improve comparability of findings, future studies should standardize leukocyte quantification using cells per milliliter of exudate (cells/mL) as the primary measurement. As mentioned, this approach reduces variability due to inconsistencies in exudate recovery and biological fluctuations in fluid volume. Moreover, this aligns with standard measurements in other inflammatory models.^98^, ^99^ For analysis of inflammatory markers, samples should be frozen for subsequent assay‐based quantification.^18^, ^23^ As mentioned, a standardized approach should focus on measuring IL‐1β, TNF‐α and IL‐6 concentrations, as these markers provide key insights into the inflammatory response in crystal arthropathies.^1^, ^9^, ^11^ Dissection of the pouch for histological assessment at the end of the protocol provides an additional useful endpoint for measuring inflammation and response to therapeutics within the pouch.^41^ Standardizing methodological considerations across these three endpoints will enhance reproducibility and improve the translational relevance findings.

As highlighted, the air pouch model can serve as a valuable guide for translational human studies that is both reproducible and standardized.^25^ Moreover, the pseudo‐membrane formed closely resembles the true synovial lining, enabling understanding of complex inflammatory interactions between immune cells, synovial macrophages, fibroblasts, chondrocytes, and endothelial cells.^24^, ^91^ However, a key limitation of the model is the absence of mechanical stress and joint movement, which may restrict its ability to accurately replicate the mechanotransduction‐driven inflammatory responses observed in crystal arthropathies.^100^ The model also mainly replicates acute inflammation rather than chronic inflammatory joint destruction and tissue remodeling characterized by recurrent gout flares.^2^, ^3^ Lastly, species differences in immune and metabolic responses may impact the translatability of findings.^95^, ^96^ Despite this, the model provides a reliable and controlled system for early evaluation of potential therapeutics targeting the MSU and CPP crystal‐mediated acute immune responses. Future research should focus on validating key inflammatory pathways and therapeutic targets identified in the model through clinical studies in human patients. Future studies should also incorporate multiple models to examine different aspects of gout and pseudogout pathophysiology. The MSU peritonitis model, whilst also lacking the anatomical specificity of the joint, provides a rapid and controlled platform for investigating crystal‐mediated inflammatory pathways.^98^ The intra‐articular injection model, though more technically challenging and less controlled compared to the subcutaneous pouch model, may allow for the analysis of more complex crystal‐mediated interactions within the joint microenvironment.^25^ The uricase‐knockout (Uox−/−) model has been used to study hyperuricemia‐induced MSU deposition,^101^ while the K/BxN serum transfer model has provided insight into systemic arthritis.^102^ Humanized mouse models may further enhance translational relevance by incorporating human immune components. Lastly, the collagen‐induced arthritis and carrageenan‐induced air pouch, peritonitis and paw oedema models may also serve as effective comparators for inflammatory responses.^103^, ^104^, ^105^ Refining these models to better replicate human disease and integrating their findings is important for advancing knowledge and development of therapeutics targets for crystal arthropathies.

## 5 CONCLUSION

In conclusion, this is the first article to systematically review monosodium urate and calcium pyrophosphate crystal‐induced inflammation in the subcutaneous air pouch model whilst providing valuable recommendations for methodical approaches. A summarized protocol has been provided in Protocol S2 in supporting information. The model has demonstrated its reliability and effectiveness in the study of acute inflammation. Nevertheless, the significant variability in methods across studies challenges the establishment of a standardized framework for comparisons. The authors encourage future investigators employing this model to standardize their methods in accordance with the authors' recommendations, including utilizing clinically relevant crystal preparations, generation of the air pouch, stimulation of inflammation, sampling methods and choice of appropriate controls. With these considerations in mind, the subcutaneous air pouch model will continue to contribute valuable insights into the mechanisms underlying MSU and CPP crystal‐induced inflammation and inform the development of new therapeutic interventions for crystal arthropathies. Moreover, greater standardization will improve transparency and reproducibility when using the model and will enable direct comparisons between studies from different research groups.

## AUTHOR CONTRIBUTIONS

**Wenu Hewage:** Conceptualization; data curation; formal analysis; funding acquisition; investigation; methodology; project administration; software; validation; visualization; writing – original draft; writing – review and editing. **Josif Vidimce:** Writing – review and editing. **Ryan G. Shiels:** Writing – review and editing. **Michael Morgan:** Writing – review and editing. **Andrew C. Bulmer:** Conceptualization; resources; supervision; writing – original draft; writing – review and editing.

## FUNDING INFORMATION

No external funding was received to support this study.

## CONFLICT OF INTEREST STATEMENT

The authors declare that they have no competing financial interests or personal relationships that could have appeared to influence the work reported in this paper.

## ETHICS STATEMENT

Not applicable.

## Supporting information


Data S1.

## Data Availability

Data will be made available on request.
