# Supplementary material for: Systematic review of the subcutaneous air pouch model using monosodium urate and calcium pyrophosphate and recommendations for studying crystal‐related arthropathies
Source: Animal Model Exp Med. 2025 Jul 11;8(9):1611–27. doi: 10.1002/ame2.70058 (PMC12531122; doi:10.1002/ame2.70058)
Supplement: Supplementary file 1 — Data S1. [file AME2-8-1611-s001.zip › Revision Feb 2025 Supplementary Material 1.docx]

**Supplementary Material**

**Table S1. Review of Methods for Generation and Performance of the Monosodium Urate Crystal Air Pouch Model and Endpoints**

| Entry Number | Author | Group Size | Animal (age) | Pouch Generation | | Pouch Stimulation Time Point, Number of Crystals and Suspension volume | Data Collection | | Analysis | |
| --- | --- | --- | --- | --- | --- | --- | --- | --- | --- | --- |
|  |  |  |  | Protocol Length | Air Pouch Inflation Time Points and volume |  | Pouch Fluid Harvesting Technique | Measurement Time Points (Post MSU delivery) | Cell Counts | Analytes |
| 1 | Chiu | 4-5 per group | BALB/c Mouse (6–8-week-old) | 6 Days | Day 0 - 2 mL  Day 3 – 3 mL | Day 6 – 3 mg, 2 mL Saline | Animals sacrificed, 2 mL Saline injected into pouch and withdrawn | 1, 3 and 5 Hours | Leukocytes  5 Hours ~ 3.8 x 10^6^ /mL | IL-1β   3 Hours – 95 pg/mL   5 Hours– 130 pg/mL   IL-6  3 Hours - 5000 pg/mL   4 Hours -2000 pg/mL  TNF-α   3 Hours – 60 pg/mL |
| 2 | McWherter | 8-10 per group | C57BL/6J Mouse (age not specified) | 6 Days | Day 0– 5 mL  Day 3 – 3 mL | Day 6 – 20 mg, 5 mL Saline | Animals sacrificed, 5 ml heparinised Saline injected into pouch and withdrawn | 4 Hours | Leukocytes  4 Hours ~ 7.5 ± 6.5 x 10^5^ /pouch | IL-1β   4 Hours ~ 125± 25 pg/mL   IL-6   4 Hours ~ 420 ± 60 pg/mL  CXCL-1   4 Hours ~ 630 ± 170 pg /mL |
| 3 | Shiels | 3-6 per group | Wistar Rat (10-12 week) | 8 Days | Day 0 – 20 mL  Day 4 – 10 mL | Day 6 – 25 mg, 5 mL Saline | 5 mL Saline injected into pouch and 4 mL withdrawn | 1, 6, 12, 24 and 48 Hours | Leukocytes  1 Hours ~ 2.0 x 10^6^ /mL  6 Hours ~ 4.5 x 10^6^/mL  12 Hours ~18.0 x 10^6^/mL  24 Hours ~19.0 x 10^6^/mL (Peak)  48 Hours ~12.0 x 10^6^/mL | IL-6  6 Hours– 25,000 pg/mL   MCP-1  6 Hours– 17,500 pg/mL   GM-CSF  6 Hours - 13 pg/mL  IL-18  6 Hours – 80 pg/mL |
| 4 | Yang | 3-5 per group | C57BL/6J Mouse (8-10 week) | 7 Days | Day 0 – 5 mL  Day 3 – 3 mL  Day 5 – 3 mL | Day 7 – 3 mg, 1 mL PBS | Technique not specified. | 0, 4 and 8 Hours | Leukocytes  0 Hours ~ 4.5 x 10^6^/pouch  4 Hours ~ 6.0 x 10^6^/pouch  8 Hours ~10.0 x 10^6^/pouch | IL-1β   0-8 Hours ~38 pg/mL   TNF-α (mRNA)   4 Hours – 2.3-fold |
| 5 | Paré | >5 per group | CD-1 Mouse (6-8 weeks) | 8 Days | Day 0 – 3 ml  Day 3 - 3 mL | Day 7 – 1.5 mg, 1 mL PBS | Animals sacrificed, pouch dissected, and washed once with 2 mL PBS-5mM EDTA and once with 1 mL PBS-5mM EDTA | 7 Hours | Leukocytes  7 Hours ~ 1.7 x 10^6^/pouch | Not Measured |
| 6 | Pessler | 4-5 Per group | BABLB/c Mouse (6-8 weeks) | 8 Days | Day 0 – 3 mL  Day 3 – 2 mL | Day 6 – 2 mg, 1 mL PBS | Animals sacrificed, pouch dissected and washed with 2mL PBS | 1,4,9,18,27 and 50 Hours | Leukocytes  0 Hours ~ 0.4 x 10^6^ /pouch  1 Hours ~ 0.5 x 10^6^ /pouch  4 Hours ~ 4.5 x 10^6^ /pouch  6 Hours ~ 10.0 x 10^6^ /pouch (Peak)  18 Hours ~ 2.5 x 10^6^ /pouch  27 Hours ~ 2.0 x 10^6^ /pouch  50 Hours ~ 1.0 x 10^6^ /pouch | IL-6   9 Hours – 380 pg/mL  Pouch Membrane (mRNA expression)  IL-1β   4 Hours ~125 fold (Peak)  IL-6   1 Hours ~110 fold (Peak)  TNF-α   1 Hours ~115 fold (Peak) |
| 7 | Iverson | 10 per group | Sprague Dawley Rat (age not specified) | 6 Days | Day 0 – 30 mL | Day 6 – 150 mg, 15 mL PBS | Technique not specified. | 4 Hours | Leukocytes  4 Hours ~ 8 x 10^6^ /mL | IL-1β –   4 Hours -7.6 ng/mL  PGE2-   4 Hours - 40 ng/mL |
| 8 | Yao | 8 Per group | ICR Mouse (age not specified) | 6 Days | Day 0 – 5 mL  Day 3 – 3 mL | Day 6 – 3 mg, 1 mL PBS | Technique not specified. | 6 Hours | Leukocytes   6 Hours – 14.39 ± 1.80 x 10^6^ /pouch,  Neutrophils: 91% | IL-1β   6 Hours ~ 90 pg/mL  IL-6   6 Hours ~ 130 pg/mL  TNF-α   6 Hours ~ 480 pg/mL |
| 9 | Yang QB | 4-5 per group | C57BL/6 Mouse (8-10 weeks) | 8 Days | Day 0 – 5 mL  Day 3 – 3 mL  Day 5 – 3 mL | Day 7 – 3 mg, 1 mL PBS | Animals sacrificed at each time point. Technique not specified. | 3, 6 12 or 24 Hours | Leukocytes  3 Hours ~ 2.0 x 10^6^/pouch  6 Hours ~ 5.5 x 10^6^/pouch  12Hours ~11.0 x 10^6^/pouch (peak)  24 Hours ~7.5 x 10^6^/pouch | IL-1β   3 Hours ~ 280pg/mL  6 Hours ~ 370pg/mL |
| 10 | Yang | 5 Per group | C57BL/6 Mouse (7-8 weeks) | 6 Days | Day 0 – 5 mL  Day 3 – 5 mL | Day 6 – 3 mg, 1 mL PBS | Technique not specified. Harvested using 2 mL PBS-5 mM EDTA. | 6 Hours | Not Measured | IL-1β   6 Hours ~750pg/mL  MPO Activity  6 Hours ~32.5 mU/mL |
| 11 | Qiao | 5 per group | C57BL/6 Mouse (8-10 weeks) | 3 Days | Day 0 - 5 mL Day 1 - 3 mL | Day 3 – 3 mg, 1 mL PBS | Technique not specified. Harvested using 2 mL PBS-5 mM EDTA. | 6 Hours | Not Measured | IL-1β  6 Hours ~385pg/mL |
| 12 | Rull | 4 per group | Sprague Dawley Rat (age not specified) | 13 Days | Day 0 – 10 mL  Day 2-6 – Air given every 2 or 3 days when needed to maintain inflation (Volume/criteria not specified) | Day 6 – 15 mg, Saline (volume not specified) | Not specified | 6, 24, 48 Hours and 7 Days | Leukocytes   6 Hours - 1462 /mm^3^  24 Hours - 1200 /mm^3^  48 Hours - 1225/mm^3^ (Peak)  7 Days Hours - 666 /mm^3^ | Not Measured |
| 13 | Laure Campillo-Gimenez | Not specified | C57BL/6J Mouse (8 Weeks) | 7 Days | Day 0 – 3ml Day 3 – 3ml | Day 6 - 1 mg/mL in PBS (amount of crystals and suspension volume not specified) | Animal sacrificed, pouch dissected and washed twice with 2 mL PBS | 6 and 24 Hours | Leukocytes   6 Hours ~1 x 106 /pouch, Neutrophils: 63 ± 1%)  24 Hours ~0.8 x 106 /pouch Neutrophils: 45 ± 2%) | IL-1β   6 Hours ~ 10 pg/mL  24 Hours – >10 pf/mL  CXCL-1-   6 Hours ~ 40 pg/mL)  24 Hours - >10 pg /mL |
| 14 | Wang | 3-4 per group | C57BL/6J Mouse (8-12 weeks) | 7 Days | Day 0 – 5 mL  Day 4 – 3 mL  Day 5 – 3 mL | Day 7 – 3 mg, 1mL (amount of crystals and suspension volume not specified) | Animal sacrificed, pouch dissected and washed with 2 mL PBS | 3,6, and 12 Hours | Leukocytes  3 Hours - 1.5 x 10^6^/pouch  6 Hours - 4 x 10^6^/pouch  12 Hours - 6 x 10^6^/pouch | IL-1β   3 Hours - 400 pg/mL (Peak)  IL-4  3 Hours - 45 pg/mL (Peak) |
| 15 | Tate | 12 per group | Sprague Dawley Rat (age not specified) | 6 Days | Day 0 – 20 mL | Day 6 – 50 mg, 5 mL Saline | Animal sacrificed, pouch dissected, and exudate directly aspirated. | 6 Hours | Leukocytes  6 Hours ~ 30000 /mm3 | Not Measured |
| 16 | Nalbant | 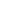  \| 8 per group \| \| --- \| | Sprague Dawley Rat (age not specified) | 7 Days | Day 0 - 24 mL  Day 3 - Given selectively (volume and criteria not specified) | Day 6 – 10 mg, 10 mL Saline | 5 mL Saline injected into pouch and withdrawn | 24 Hours | Leukocytes  24 Hours -1412.5 ± 039.7 /mm3 | TNF-α   24 Hours -0.79 ± 0.05 ng/mL |
| 17 | Murakami | 10 per group | C57BL/6 Mouse (6-8weeks) | 6 Days | Day 0 – 5mL  Day 3 – 3 mL | Day 5 – 3 mg, 1 mL PBS | 3 ml cold PBS injected into pouch and withdrawn | 0,4,8,12 and 24 Hours | Leukocytes  0 Hours ~ 0.4 x 10^6^ /pouch  4 Hours ~ 2.0 x 10^6^ /pouch  8 Hours ~ 4.7 x 10^6^ /pouch (peak)  12 Hours ~ 2.0 x 10^6^ /pouch  24 Hours ~ 2.0 x 10^6^ /pouch | CXCL1  4 Hours - 3.5 ng/pouch (Peak) |
| 18 | Ortiz-Bravo | 4 per group | Sprague Dawley Rat (age not specified) | 9 Days | Day 0 – 20 mL  Day 3 – 10 mL | Day 6 – 20 mg, 5 mL Saline | Fluid directly aspirated from pouch. | 1, 6, 24, 48 and 72 Hours | Leukocytes  1 Hours - 162 ± 51.5 /mm^3^  6 Hours - 21,062 ± 476.7 /mm^3^ (Peak)  24 Hours - 10,400 ± 1.034 /mm^3^   48 Hours - 7,462 ± 3,910 /mm^3^   72 Hours - 4,650 ± 1,800 /mm^3^ | Not Measured |
| 19 | Hsu | 6 per Group | Sprague Dawley Rat (age not specified) | 6 Days | Day 0 – 24 mL  Day 3 – Given selectively if necessary (Volume/criteria not specified) | Day 6 – 5 mg/Rat in PBS (amount of crystals and suspension volume not specified) | 5 mL of PBS injected into the pouch, instilled and withdrawn three times | 6, and 12 Hours | Leukocytes  6 Hours ~ 50 x 10^6^ /pouch  12 Hours ~ 52 x 10^6^ /pouch | IL-1β   12 Hours ~ 180 pg/pouch  TNF-α   12 Hours ~ 3900 pg/pouch  IL-6   12 Hours ~ 23000 pg/pouch |
| 20 | Desai | 5 per group | C57BL/6 Mouse (6 weeks) | Not specified | Not specified | 2.5 mg, PBS (suspension volume and date of administration not specified) | Technique not specified. | 24 Hours | Neutrophils   24 Hours ~ 8000 /µL | N/A |
| 21 | Ryckman | >7 Per Group | CD1 or BALB/c Mouse (10-12 week) | 8 Days | Day 0 – 3 ml  Day 3 - 3 mL | Day 7 – 1.5 mg, 1 mL PBS | Animal sacrificed, pouch dissected, washed once with 1 mL PBS-5mM EDTA and twice with 2 mL PBS-5mM EDTA | 0, 3, 6, 9, 12, 24 Hours | Leukocytes  0 Hours ~ 0.1 x 10^6^ /pouch  3 Hours ~ 0.6 x 10^6^ /pouch  6 Hours ~ 2.4 x 10^6^ /pouch  9 Hours ~ 3.8 x 10^6^ /pouch (peak)  12 Hours ~ 2.7 x 10^6^ /pouch  24 Hours ~ 0.8 x 10^6^ /pouch | CXCL1   3 Hours -550 ± 145 pg (Peak)  CXCL2   3 Hours - 1800 ± 170 pg (Peak)  MCP1  3 Hours -13500 ± 3950 pg/mL (Peak)   CCL3  8 Hours -1290 ± 170 pg/mL (Peak) |
| 22 | Reber | 8-9 per group | C57BL/6 Mouse (age not specified) | 6 Days | Day 0 – 3 mL  Day 3 – 3 mL | Day 6 – 3 mg, 1 mL PBS | Technique not specified. | 6 Hours | 6 Hour  Leukocyte ~ 6 x 10^6^ /pouch  Neutrophils ~ 5 x 10^6^ /pouch  Eosinophil ~ 0.5 x 10^6^ /pouch  Monocyte ~ 0.3 x 10^6^ /pouch | IL-1β   6 Hours ~ 150 pg/mL |
| 23 | Yang | 5 Per group | C57BL/6 Mouse (7-8 weeks) | 6 Days | Day 0 – 5 mL  Day 3 – 5 mL | Day 4 – 3 mg, 1 mL PBS | Technique not specified. Harvested using 2 mL PBS in 5 mM EDTA | 6 Hours | Not collected | IL-1β   6 Hours ~ 1.25 ng/mL  IL-18  6 Hours ~110 pg/mL  Caspase-1 Activity  6 Hours - 3-fold |
| 24 | Moilanen | 5-8 per group | B6;129P/J Mouse (age not specified) | 7 Days | Day 0 – 3 mL  Day 2 - 1.5 mL | Day 7 – 3 mg, 1 mL PBS | Technique not specified. | 6 Hours | Leukocytes  6 Hours - 1.205 ± 0.211 x 10^6^ | IL-1β   6 Hours - 37.6 ± 5.0 pg/ pouch   IL-6   6 Hours - 184.1 ± 41.2pg / pouch   MCP-1  6 Hours - 1946.6 ± 396.0 pg / pouch   MIP-1α  6 Hours - 142.2 ± 33.6 pg / pouch   MIP-2  6 Hours - 256.1 ± 78.5 pg / pouch |
| 25 | Lee | 3-6 per group | C57BL/6 Mouse (7-8 weeks) | 4 Days | Day 0 – 5 ml  Day 3 – 5 mL | Day 4 – 3 mg/mL in PBS (suspension volume not specified) | Technique not specified. Harvested using 2 mL PBS in 5 mM EDTA. | 6 Hours | Not collected | IL-1β   6 Hours ~1250 pg/mL  IL-18  6 Hours ~100 pg/mL  Caspase-1 Activity  6 Hours ~ 3.4 x relative to control   MPO Activity  6 Hours ~ 32.5 mU/mL |
| 26 | Hsu | 6 per group | Sprague Dawley Rat (age not specified) | 6 Days | Day 0 – 24 mL  Day 3 – Given selectively if necessary (Volume/criteria not specified) | Day 6 - 5 mg/Rat in PBS (amount of crystals and suspension volume not specified) | 5 mL of PBS was injected into the pouch, instilled and withdrawn three times. | 6, 12 Hours | Leukocytes  6 Hours ~ 50 x 10^6^ /pouch  12 Hours ~ 52 x 10^6^ /pouch | IL-1β   12 Hours ~ 180 pg/pouch  TNF-α   12 Hours ~ 3900 pg/pouch  IL-6   12 Hours ~ 23000 pg/pouch |
| 27 | Lioté | 5-10 per group | Sprague Dawley Rat (age not specified) | 10 Days | Day 0 – 20 mL  Day 3 – 10 mL | Day 7 – 5 mg, 5 mL Saline | Pouch catheterised. Technique not specified. | 0.5, 1, 6, 24. 48 and 72 Hours | Leukocytes   0.5 Hours ~ 800 /mm^3^  3 Hours ~ 600/ mm^3^  6 Hours ~ 9500/mm^3^(Peak)  24 Hours ~ 5000 /mm^3^  48Hours ~ 1800/ mm^3^  72 Hours ~ 400/mm^3^ | Not Measured |
| 28 | Brooks | 6 per group | Wistar Rat (age not specified) | 7-10 Days | Day 0 – 15 mL  Day 2 - 15 mL  Day 4 – 15 mL | Day 7-10 – 150 mg, 15 mL Saline (Administered via 18G dwelling catheter) | 1 mL aspirated via 18G indwelling catheter and collected into EDTA (10 mM) | 1,2,4,6, and 8 Hours | Rate of Leukocyte infiltration  0 Hours ~ 0.02 x 10^6^/ml/hr   1 Hours ~ 0 x 10^6^/ml/hr  2 Hours ~ 0.25 x 10^6^/ml/hr  4 Hours ~ 1.05 x 10^6^/ml/hr  6 Hours ~ 1.15 x 10^6^/ml/hr (Peak)  8 Hours - 0.4 x 10^6^/ml/hr | Rate of Plasma Extravasation   6 Hours ~ 5.5. x 10-2 AU /hr (Peak)  LTB4  6 Hours ~ 14 nM (Peak) |
| 29 | Terkeltaub | 7-15 per group | C57BL/6 Mouse (14 weeks) | 7 Days | Day 0 – 5 mL  Day 4 – 3 mL | Day 7 – 10 mg, 1 mL Saline | Animal sacraficed, pouch dissected and washed with 1 mL PBS-0.0005% phenol red- 20 units/mL heparin | 4 Hours | Leukocytes  4 Hours – 750 ± 623 /mm^3^ | Not Measured |
| 30 | Wang | 15 per group | C57BL/6/129 (6-8 weeks) | 7 Days | Day 0 – 5 ml  Day 3 – 3 mL | Day 7 – 3 mg, 1 mL PBS | Not specified | 6 Hours | Leukocytes  6 Hours ~7.5 x 10^6^ /pouch | IL-1β   6 Hours ~ 2500 pg/pouch   CXCL-1-   6 Hours ~ 4500 pg/pouch |
| 31 | Ru Liu-Bryan | 8-9 per group | C57BL/6 Mouse (10-12 weeks old) | 8 Days | Day 0 – 5 ml  Day 3 – 3 mL | Day 7 – 3 mg, 1 mL PBS | 5 mL PBS containing 5 mM EDTA injected into pouch and wirhdrawn | 0, 6 24 Hours | Leukocytes  0 Hours ~ 0.5 x 10^6^/pouch  6 Hours ~ 13 x 10^6^ /pouch (Peak)  24 Hours ~ 2 x 10^6^ /pouch | IL-1β   0 Hours - 3 pg/mL   6 Hours - 330 pg/mL (Peak)  24 Hours - 10 pg/ mL |
| 32 | Murakami | 4-6 per group | C57BL/6 Mouse (6-8 weeks) | 6 Days | Day 0 – 5mL  Day 3 – 3 mL | Day 7 – 3 mg, (amount of crystals, ssupension solution and suepension volume not specified) | 1mL cold PBS-0.005% phenol red-20 units/mL heparin injected an withdrawn | 0,4, 8 and 12 Hours | Not measured | IL-1β   0 Hours ~50 pg/pouch  4 Hours ~225 pg/pouch  8 Hours ~ 1150 pg/pouch (Peak)  12 Hours ~ 300 pg/pouch  MIP-2  0 Hours ~  0 pg/pouch  4 Hours ~ 200 pg/pouch  8 Hours ~ 550 pg/pouch (Peak)   12 Hours ~ 200 pg/pouch |
| 33 | Schlitz | 6-8 per group | Sprague Dawley Rat (age not specified) | 8 Days | Day 0 – 22 mL Day 2 – 22 mL | Day 6 – 10 mg, 5 mL Saline | Pouch Catheterised. Method not specified. | 1, 2, 4, 6, 24 and 48 Hours | Neutrophils   4 Hours ~14/mm)   24 Hours ~27/mm)   Macrophage  2 Hours ~130/mm  Mast Cell   2 Hours ~23/mm | Histamine   1 Hours ~ 240 nM/mL  2 Hours ~ 425nM/mL (Peak)  4 Hours ~ 210 nM/mL  6 Hours ~ 50 nm/mL  24 Hours ~ 150 nM/mL  48 Hours ~ 325 nM/mL |
| 34 | Inokuchi | 5 per group | C57BL/6 Mouse (6-8 weeks) | 7 Days | Day 0 – 3 mL  Day 3 – 5 mL | Day 7 - 3 mg, PBS (suspension volume not specified) | 3 mL PBS injected into pouch and withdrawn | 8 Hours | Leukocytes  8 Hours -1.41 ± 0.42 x 10^6^  Neutrophils   8 Hours -1.28 ± 0.40 x 10^6^ | IL-18  8 Hours -Not detected  IL-1β   8 Hours -947.0 ± 595.4 pg/mL  CXCL1  8 Hours -652.9 ± 323.6 pg/mL  MIP-1α  8 Hours -52.8 ± 17.7 pg/mL  IL-6  8 Hours -553.8 ± 306.6 pg/mL |
| 35 | Scanu | 7 per group | CD1 Mouse (8-9 weeks) | 7 Days | Day 0 – 3.5 mL  Day 3 – 3.5 mL | Day 6 - 2 mg in 1mL PBS | Animal euthanised, pouch dissected and washed with 2 mL PBS | 0, 3, 6, 24 Hours | Leukocytes   0 Hours ~ 2 x 10^5^ /mL  3 Hours -8 x 10^5^ /mL (First Peak)   6 Hours ~ 2.5 x 105 /mL  24 Hours ~ 6 x 10^5^ /mL (Second Peak)   Neutrophils   0 Hours ~ 0.1 x 10^5^ /mL  3 Hours ~ 5.2 x 10^5^ /mL (First Peak)   6 Hours ~ 0.4 x 10^5^ /mL  24 Hours ~ 2.4x 10^5^ /mL (Second Peak) | IL-1β   0 Hours ~ 2 pg/mL   3 Hours ~ 38 pg/mL (Peak)   6 Hours ~ 19 pg/mL  24 Hours ~ 12 pg/mL  IL-1Ra   3 Hours ~ 200 pg/mL   IL-6   0 Hours ~ 90 pg/mL  3 Hours ~550 pg/mL (Peak)  6 Hours ~ 275 pg/mL  24 Hours ~ 90 pg/mL CXCL1  0 Hours ~ 40 pg/mL  3 Hours ~ 390 pg/mL (peak)  6 Hours ~ 130 pg/mL  24 Hours ~ 300 pg/mL  CCL2   0 Hours ~ 100 pg/mL  3 Hours ~ 1600 pg/mL  6 Hours ~ 200 pg/mL (peak)  24 Hours ~ 200 pg/mL |
| 36 | Della Beffa | 3 per group | BALB/c Mouse (6 weeks) | 8 Days | Day 0 – 3 mL  Day 3 – 2 mL | Day 6 – 2 mg, 1mL PBS | Animal euthanised, pouch dissected and washed with PBS (volume not specified.) | 1,4,9,18,27 and 50 Hours | Leukocytes   0 Hours ~ 0.2 x 10^6^ /pouch  1 Hours ~ 0.4 x 10^6^ /pouch  4 Hours ~ 4.5 x 106 /pouch  9 Hours ~ 10 x 10^6^ /pouch (Peak)  18 Hours ~ 2 x 10^6^ /pouch  27 Hours ~ 1.2 x 10^6^ /pouch  50 Hours ~ 0.8 x 10^6^ /pouch | IL-1β (mRNA)   4 Hours -208-fold (Peak)  HIF-1α  4 Hours -6.6-fold (Peak)  GAPDH   18 Hours -2.8fold (Peak)  PPIA  0-50 Hours -nonsignificant rise |
| 37 | Tate | >6 Per group | Sprague Dawley Rat (age not specified) | 6 Days | Day 0 – 20 mL | Day 6 – 1,5,10.25 and 50 mg, 5 mL Saline | Animal euthanised, pouch dissected and directly aspirated | 6 Hours | Leukocytes 6 Hour  1 mg MSU ~ 2000 /mm^3^  5 mg MSU ~ 9000 /mm^3^  10 mg MSU ~ 17500/mm^3^  25 mg MSU ~ 21000/mm^3^  50 mg MSU ~ 29000/mm^3^  Neutrophils 6 Hour  1 mg MSU ~ 1340 /mm^3^  5 mg MSU ~ 7020 /mm^3^  10 mg MSU ~ 14525/mm^3^  25 mg MSU ~ 18900/mm^3^  50 mg MSU ~ 26970/mm^3^ | Not measured |
| 38 | Ferrari | 6 per group | Sprague Dawley (age not specified) | 9 Days | Day 0 – 20 mL  Day 4 – 10 mL | Day 6 – 25 mg, 4 mL PBS | 5 mL Saline injected into pouch and withdrawn | 0, 6, 12, 24 and 72 Hours | Leukocytes   6 Hours ~ 4000/mm^3^  24 Hours ~2800/mm^3^ | TNF-α   6 Hours ~ 4 ng/mL  24 Hours ~ 32 ng/mL  PGE2  6 Hours - 7661 pg/mL  24 Hours - 125 pg/mL |
| 39 | Hoffman | 9 per group | C57BL/6 Mouse (8-10 weeks) | 8 Days | Day 0 – 5 mL  Day 3 – 3 mL | Day 7 – 3 mg, 1 mL PBS | Pouch injected with 5 mL PBS-5mM EDTA and withdrawn | 0, 6, 24 Hours | Leukocytes   0 Hours ~ 0.05 x 10^6^ /mL  6 Hours ~ 0.55 x 10^6^ /pouch (Peak)   24 Hours ~ 0.18 x 10^6^ /pouch | IL-1β   6 Hours ~ 135 pg/mL |
| 40 | Jung | 4 per group | BABLB/c Mouse (8 week) | 7 ½ Days | Day 0 - 3 mL  Day 3 – 3 mL | Day 6 – 2 mg, 1 mL PBS | Animal euthanised, pouch dissected, washed with 2 mL PBS, lavaged using pipette | 0, 1, 4, 6, 9, 12, 18, 24 and 36 Hours | Leukocytes   0 Hours ~ 0.4 x 10^6^ /pouch  1 Hours ~ 0.6 x 10^6^ /pouch  4 Hours ~ 2.0 x 10^6^ /pouch  6 Hours ~ 3.8 x 10^6^ /pouch  9 Hours ~ 7.8 x 10^6^ /pouch (Peak)   12 Hours ~ 5.6 x 10^6^ /pouch  18 Hours ~ 5.0 x 10^6^ /pouch  24 Hours ~ 3.6 x 10^6^ /pouch  36 Hours ~ 2.5 x 10^6^ /pouch  Neutrophils 9 Hours ~ 19.2 x 10^3^ /pouch (Peak) | IL-6   9 Hours -391.54 ± 16.77 pg /mL  PGE2  9 Hours -1530.49 ± 205.93 pg /mL  PGD2  9 Hours -11.02 ± 2.49 pg /mL   Pouch Membrane mRNA Expression  IL-6   9 Hours ~ 55. 47 ± 2.68-fold  TNF-α  9 Hours ~ 20.43 ± 2.91-fold |
| 41 | Torres | 5 per group | C57BL/6 Mouse (12-16 weeks) | 7 Days | Day 0 – 5 mL  Day 3 – 3 mL | Day 7 - 1 mg, 0.5 mL PBS | 5 mL stain buffer injected into pouch and withdrawn | 6 Hours | Leukocytes   6 Hours ~ 3.8 x 10^6^ /pouch | Not measured |
| 42 | Singh | 9 per group | Wistar (age not specified) | 7 Days | Day 0 – 10 mL Day 2 – 10 mL  Day 4 – 10 mL | Day 6 – 10 mg, 10 mL Saline | Pouch directed aspirated using glass syringe and placed into heparinised Saline (concentration not specified) | 6, 12 24 Hours | Not Presented for MSU only group | Not measured |
| 43 | Murakami | 3-6 per group | C57BL/6 Mouse (6-8 weeks) | 7 Days | Day 0 – 5 mL  Day 3 – 3 mL | Day 7 – 3 mg, 1mL PBS | 3 mL cold PBS injected into pouch and withdrawn | 0, 2, 4, 8 and 12 Hours | Leukocytes  0 Hours ~ 5.0 x 10^6^ /pouch  2 Hours ~ 5.0 x 10^6^ /pouch  4 Hours ~ 20.0 x 10^6^ /pouch  8 Hours ~ 100 x 10^6^ /pouch (Peak)  12 Hours ~ 30.0 x 10^6^ /pouch) | Not measured |
| 44 | Ponce | 3-4 per group | BALB/c Mouse (10 weeks) | 7 Days | Day 0 – 5 mL  Day 3 – 3 mL | Day 6 - 10 mg, 2 mL PBS | Animal euthanised, pouch dissected and washed with 2 mL PBS-20 units heparin | 2, 6, 12 and 24 Hours | Leukocytes  2 Hours -1,264,583 ± 434,771 /mL  6 Hours ~ 4,500,000 ± 500,000 /mL  12 Hours -6,071,250 ± 1,043,853/mL (Peak)  24 Hours -3,795,000 ± 11,724,824/mL) | Not measured |
| 45 | Singh | 3 per group | Wistar Rat (age not specified) | 7 Days | Day 0 – 10 mL Day 2 – 10 mL  Day 4 – 10 mL | Day 6 – 10 mg, 10 mL Saline | Pouch directly aspirated using glass syringe and placed into heparinised Saline (concentration not specified.) | 6, 12 24 Hours | Not Presented | Not measured |
| 46 | Nalbant | 5 per group | Sprague Dawley Rat (age not specified) | 7 Days | Day 0 – 24 mL  Day 2 - Given selectively (Volume/criteria not specified) | Day 6 – 5 mg, 5 mL Saline | Not specified | 24 Hours | Leukocytes  24 Hours - 2508 ± 792.3 /mm^3^ | IL-10   24 Hours -Non-significant rise  TNF-α   24 Hours - 70.34 ± 20.9 ng/mL |
| 47 | Forrest | 6-7 per group | Wistar (age not specified) | 7 Days | Day 0 – 15-20 mL  Day 3 – 15-20 mL | Day 7 - 100 mg, 10 mL PBS | Animal euthanised, pouch dissected, pouch fluid directly removed using plastic pipette | 4 Hours | Leukocytes  4 Hours ~ 6.0 x 10^6^ /mL | LTB4  4 Hours -2429 ± 227 pg /mL  PGE2  4 Hours - 12 353 ± 3995 pg /mL  6-oxo-PGF1⍺  4 Hours -34 443 ± 9749 pg /mL  TXB2  4 Hours -16 717 ± 4340 pg /mL |
| 48 | Tate | 10 per group | Sprague Dawley Rat (age not specified) | 6 Days | Day 0 – 20 mL | Day 6 – 10 mg, 5 mL Saline | Animal euthanised, pouch dissected and directly aspirated | 6 Hours | Not measured | Not measured |
| 49 | Scott | 3-9 per group | C57BL/6 (age not specified) | 8 Days | Day 0 – 5 ml  Day 3 – 3 mL | Day 7 – 3 mg, 1 mL PBS | Pouch injected with 5 mL PBS-5 mM EDTA and withdrawn | 0, 6 24 Hours | Leukocytes  0 Hours ~ 0.02 x 10^6^ /mL  6 Hours ~ 0.88 x 10^6^ /mL (Peak)  24 Hours ~ 0.2 x 10^6^ /mL  Neutrophils  0 Hours ~ 0.02 x 10^6^ /mL  6 Hours ~ 1.02 x 10^6^ /mL (Peak)  24 Hours ~ 0.2 x 10^6^ /mL | IL-1β   0 Hours ~ 0 pg/mL  6 Hours ~ 1100 pg/mL (Peak)  24 Hours ~ 50 pg/mL  CXCL1  0 Hours ~ 0 pg/mL  6 Hours ~ 1100 pg/mL (Peak)  24 Hours ~ 50 pg/mL |
| 50 | Ortiz-Bravo | 4 per group | Sprague Dawley Rat (age not specified) | 9 Days | Day 0 – 20 mL  Day 3 – 10 mL | Day 6 – 20 mg, 5 mL Saline | Pouch directly aspirated using syringe | 1, 6, 24, 48. 72 Hours | Leukocytes  1 Hours -162 ± 51.5 /mm^3^  6 Hours -21062 ± 476.7 /mm^3^ (Peak)  24 Hours -10400 ± 1034 /mm^3^  48 Hours -7462 ± 3910 /mm^3^  72 Hours -4650 ± 1800 /mm^3^ | Not measured |
| 51 | Uratsuji | 5 per group | BALB/c Mouse (8 weeks) | 7 Days | Day 0 – 3 mL  Day 3 – 3 mL | Day 7 – 2mg, 1 mL PBS | 1 mL PBS injected into pouch and withdrawn | 9 Hours | Leukocytes  9 Hours ~ 1.5 x 10^6^ cells | Not measured |
| 52 | Generini | 5 per group | Sprague Dawley Rat (6-8 weeks) | 7 Days | Day 0 – 20 mL  Day 2-6 – Air given whenever needed to maintain inflation (volume and criteria not specified) | Day 6 - 5 mg. 5 mL Saline | Not specified | 6, 12 and 24 Hours | Leukocytes  6 Hours -450 ± 250 /mm^3^  12 Hours -1250 ± 400 /mm^3^ (Peak)  24 Hours -650 ± 300 /mm^3^ | Not Measured |
| 53 | Akahoshi | 12 per group | C57BL/6 Mouse (8-12 weeks) | 8 Days | Day 0 – 3 mL air  Day 3 – 5 mL | Day 7 – 3 mg, 1 mL PBS | 3 mL PBS injected and withdrawn | 4, 8 and 12 Hours | Leukocytes  4 Hours -2.25 x 10^6^ cells  8 Hours -4.25 x 10^6^ cells  12 Hours -2.25 x 10^6^ cells | Not Measured |
| 54 | Sedgwick | >6 per group | Wistar Rat (age not specified) | 2/4/7 Days | Day 0 – 20 mL Air Day 2-5 - 10 mL air given every 3 days to maintain inflation (criteria not specified) | Day -1 or -3 or -6 – 0.1- or 1- or 5- or 10- mg, 1- or 5-mL Saline | Pouch directly aspirated | 2, 4, 6 and 24 Hours | Leukocytes  6 Day Pouch, 4 Hours , 0.1 mg Crystals  1 mL suspension volume ~ 5 x 10^6^  cells  5 mL suspension volume ~ 10 x 10^6^ cells  6 Day Pouch, 4 Hour,1.0 mg Crystals  1 mL suspension volume ~ 15 x 10^6^ cells  5 mL suspension volume ~ 16 x 10^6^ cells  6 Day Pouch, 4 Hour, 5.0 mg Crystals  1 mL suspension volume ~ 16 x 10^6^ cells  5 mL suspension volume ~ 60 x 10^6^ cells  6 Day Pouch, 4 Hour,10.0 mg Crystals  20 mL suspension volume ~ 20 x 10^6^ cells  5 mL suspension volume ~ 100 x 10^6^ cells  1 Day pouch, 10 mg crystals in 5 mL  2 Hour~ 10 x 10^6^ cells  4 Hour~ 10 x 10^6^ cells  6 Hour~ 10 x 106 cells  24 Hour~ 10 x 10^6^ cells  3 Day pouch, 10 mg crystals in 5 mL  2 Hour~ 10 x 106 cells  4 Hour~ 50 x 10^6^ cells  6 Hour~ 10 x 106 cells  24 Hour~ 10 x 10^6^ cells  6 Day pouch, 10 mg crystals in 5 mL  2 Hour~ 10 x 10^6^ cells  4 Hour~ 90 x 10^6^ cells  6 Hour~ 150 x 10^6^ cells  24 Hour~ 155 x 10^6^ cells | Not measured |
| 55 | Rossetti | 19 per group | Sprague Dawley Rat (age not specified) | 6 Days | Day 0 – 20 mL | Day 6 – 10 mg, 2 mL Saline | Anima euthanised, pouch dissected, and fluid directly aspirated | 6 Hours | Leukocytes  6 Hours -11.8 x 10^3^ /mm3 | Not measured |
| 56 | Gordon | 4 per group | Wistar Rat (age not specified) | 7 Days | Day 0 – 20 mL  Day 3 – 10 mL | Day 7 – 20 mg, 5 mL Saline | 5mL Saline injected into pouch and withdrawn | 3 and 6 Hours | Leukocytes  3 Hours -5.6 ± 1.3 x 10^6^ cells  6 Hours -56.2 ± 12.1 x 10^6^ cells | 6-Keto-PGF1⍺  3 Hours -12.4 ± 1.3 ng/mL  6 Hours -18.7 ± 5.2 ng/mL |
| 57 | Choi | 5 per group | C57BL/6 Mouse (8 weeks) | 4 Days | Day 0 – 5 mL  Day 3 – 5 mL | Day 4 – 3 mg, 1 mL PBS | Pouch injected with 2 mL PBS-5 mM EDTA and withdrawn | 6 Hours | Not Measured | IL-1β   6 Hours ~ 330 ± 10 pg/mL  IL-18   6 Hours ~ 150 pg/mL  MPO Activity   6 Hours ~ 4 ± 2 mU/mL |
| 58 | Wang | Not specified | BABLB/c Mouse (8 weeks) | 6 Days | Day 0 – 3 mL  Day 3 – 2mL | Day 6 – 2 mg, 0.5 mL PBS | Animal euthanised, pouch dissected and fluid directly aspirated | 6 Hours | Not Measured | IL-1β   6 Hours ~ 225 ± 25 pg/mL |
| 59 | Stubelius | 3-5 per group | C57BL/6 Mouse (7-9 weeks) | 6 Days | Day 0 – 3mL  Day 3 – 3mL | Day 6 – 3 mg, 0.5 mL PBS | Pouch injected with 3 mL PBS, pouch massaged for 30 sec and withdrawn | 8 Hours | Leukocytes  8 Hours ~ 3.5 ± 0.5 x 10^6^ cells/mL | IL-1β   6 Hours ~ 320 ± 40 pg/mL  CXCL1  6 Hours ~ 600 ± 180 pg/mL |
| 60 | Yang | 4-6 per group | C57BL/6 Mouse (8-10 weeks) | 8 Days | Day 0 – 5mL | Day 7 – 3 mg (suspension solution and volume not specified) | Technique not specified. | 3,6,12,24 Hours | Leukocytes  3 Hours ~ 1.5 x 10^6^ cells  6 Hours ~ 2.5.0 x 10^6^ cells  12 Hours ~ 3.0 x 10^6^ cells 24 Hours ~ 4.0 x 10^6^ cells | IL-1β   3 Hours ~ 150 pg/mL (Peak)  6 Hours ~ 50 pg/mL  12 Hours ~ 50 pg/mL  24 Hours ~ 40 pg/mL  IL-6   3 Hours ~ 2500 pg/mL  6 Hours ~ 4000 pg/mL (Peak)  12 Hours ~ 750 pg/mL  24 Hours ~ 700 pg/mL  MCP-1  3 Hours ~ 460 pg/mL  6 Hours ~ 480 pg/mL (Peak)  12 Hours ~ 475 pg/mL  24 Hours ~ 470 pg/mL |
| 61 | Gupta | 5 per group | C57BL/6 Mouse (age not specified) | 6 Days | Day 0 – 3mL Day 3 – 3mL | Day 6- 3 mg/mL in PBS (amount of crystals and suspension volume not specified) | Technique not specified. | 6 Hours | Not Presented | IL-1β   ~ 0.8 ± 0.2 pg/mL IL-6   ~ 8 ± 2 pg/mL |
| 62 | Huang | 3-5 per group | C57BL/6 Mouse (6-8 weeks) | 7 Days | Day 0 – 3mL  Day 3 – 3 mL | Day 7 – 2 mg, 1ml PBS | Animal euthanised, pouch dissected and directly aspirated | 6 Hours | Leukocytes ~ 8 ± 1 x 10^6^ cells | IL-1β   ~ 60 ± 15 pg/mL  CXCL1  ~ 1150 ± 150 pg/mL  MCP-1  ~ 1100 ± 200 pg/mL |
| 63 | Pan | Not specified | B6 Mouse (age not specified) | 8 Days | Day 0 – 3mL  Day 3 - 2mL | Day 7 – 3 mg, 1 mL PBS | 2mL PBS injected into pouch and withdrawn | 0,6,24 Hours | Leukocytes   0 Hours ~ 5x10^5^ cells   6 Hours ~ 35 ± 5 x 10^5^ cells  Macrophages   0 Hours ~ 2x10^5^ cells   6 Hours ~ 6 ± 1x 10^5^ cells   Neutrophils   0 Hours ~ 1x10^5^ cells   6 Hours ~ 24± 6x 10^5^ cells | IL-1β   0 Hours ~ 0 pg/mL   6 Hours ~ 600 ± 200 pg/mL (peak)  24 Hours ~ 50 pg/mL  IL-6  0 Hours ~ 100 ± 100 pg/mL   6 Hours ~ 1400 ± 500 pg/mL (peak)  24 Hours ~ 50 pg/mL  MCP-1   0 Hours ~ 100 ± 25 pg/mL   6 Hours ~ 500 ± 100 pg/mL (peak)  24 Hours ~ 40 ± 40 pg/mL  CXCL1  0 Hours – 0 pg/mL   6 Hours ~ 1400 ± 50 pg/mL  CXCL2   0 Hours – 0 pg/mL   6 Hours ~ 350 ± 200 pg/mL |
| 64 | Ali | 5 per group | BABLB/c Mouse (age not specified) | 6 Days | Day 0 – 4 mL  Day 3 – Volume given not specified | Day 6 – 3 mg/ml (amount of crystals and suspension volume not specified) | Animal euthanised, pouch dissected and directly aspirated | 6 Hours | Not measured | IL-1β   6 Hours ~ 2 pg/mL   TNF-α  6 Hours ~ 1.5 pg/mL   IL-6  6 Hours ~ 4.5 pg/mL |
| 65 | Shu-Min | 3 per group | ICR Mouse (7 weeks) | 8 Days | Day 0 – 5mL  Day 3 – 3mL  Day 5 – 3mL | Day 7 – 1 mg, 0.5 mL PBS | Technique not specified | 24 Hours | Not presented | Not Measured |
| 66 | Kotiw | 5-12 per group | Sprague Dawley Rat (age not specified) | 8 Days | Day 0 – 20 mL  Day 4 – 10 mL | Day 6 – 25 mg, 5 mL Saline | Pouch injected with 5 mL Saline and 4 mL withdrawn | 1, 6, 12, 24, 48 Hours | Leukocytes 12 Hours -1.50 ± 0.10 x 10^7^ /pouch, Neutrophils:95% | TNF-α   6 Hours -450 pg/mL (Peak)  IL-6   6 Hours -27.4 ± 3.1 ng/mL (Peak) |
| 67 | Xu | 6 per group | C57BL/6 Mouse (8 weeks) | 6 Days | Day 0 – 3 mL  Day 3 – 3mL | Day 6 - 1 ml HBSS + 200 uL 25 mg/kg MSU | Pouch injected twice with 3 mL HBSSS buffer and withdrawn | 6 Hours | Neutrophils 6 Hours -16 ± 1.0 x 10^5^/ pouch | Not Measured |
| 68 | Mohapatra | 4 per group | C57BL/6 Mouse (6 weeks) | 8 Days | Day 0 – 5mL  Day 4 – 5 mL | Day 7 – 3mg, 300 uL PBS | Pouch injected with PBS (unspecified volume) and withdrawn | 24 Hours | Leukocytes 24 Hours - 5.0 ± 0.5 x 10^6^ | TNF-α   24 Hours - 75 ± 5 pg/mL  IL-6   24 Hours - 420 ± 30 pg/mL |
| 69 | Ma | 3-6 per group | 129SvEv:C57BL/6 Mouse (8 weeks) | 6 Days | Day 0 – 3 ml  Day 3 – 2 mL | Day 5- 5 mg in PBS (volume not specified) | Technique not specified. | 12 Hours | Not presented | Not presented |
| 70 | Huang | 16 per group | C57BL/6 Mouse (8 weeks) | 8 Days | Day 0 - 4 mL  Day 3 - 3 mL | Day 6 - 3 mg in 1 mL in PBS | Pouch injected with 1 mL PBS and withdrawn | 0,4,8,16, 24, 48 Hours | Not presented | IL-1β   0 Hours ~ 10 pg/mL   4 Hours ~ 35 pg/mL   8 Hours ~ 45 pg/mL (peak)  16 Hours ~ 25 pg/mL   24 Hours ~ 25 pg/mL   48 Hours ~ 15 pg/mL  TNF-α   0 Hours ~ 7.5 pg/mL   4 Hours ~ 50 pg/mL   8 Hours ~ 45 pg/mL   16 Hours ~ 35 pg/mL  24 Hours ~ 75 pg/mL (peak)   48 Hours ~ 17.5 pg/mL  MCP-1  0 Hours ~ 200 pg/mL   4 Hours ~ 400 pg/mL   8 Hours ~ 350 pg/mL   16 Hours ~ 600 pg/mL (peak)   24 Hours ~ 500 pg/mL   48 Hours ~ 200 pg/mL  IL-6  0 Hours ~ 25 pg/mL   4 Hours ~ 140 pg/mL   8 Hours ~ 125 pg/mL   16 Hours ~ 225 pg/mL (peak)   24 Hours ~ 100 pg/mL   48 Hours ~ 75 pg/mL |
| 72 | Yan | 4-6 per group | C57BL/6 Mouse (8 weeks) | 8 Days | Day 0 – 5 mL  Day 3 – 3 mL | Day 7 - 3 mg, 1 mL PBS | Pouch injected with 3 mL PBS containing 25 units/mL heparin and 10% foetal bovine serum and withdrawn | 4, 18, 12 Hours | Leukocytes  0 Hours ~ 5.5 x 10^6^/pouch | Not Measured |
| 73 | Hemstapat | 6 per group | Wistar Rat (7-8 weeks) | 7 Days | Day 0 - 20 mL  Day 3 - 10 mL | Day 6 - 15 mg, 10 mL HEPES buffered saline | Technique not specified. | 24 Hours | Leukocytes  4 Hours ~ 1.25 x 10^6^ cells  8 Hours ~ 1.5 x 10^6^ cells  12 Hours ~ 3.0 x 10^6^ cells | IL-1β   4 Hours ~ 50 pg/mL   8 Hours ~ 100 pg/mL (peak)  12 Hours ~ 75 pg/mL   TNF-α   4 Hours ~ 450 pg/mL   8 Hours ~ 600 pg/mL (peak)  12 Hours ~ 500 pg/mL  IL-18  4 Hours ~ 65 pg/mL   8 Hours ~ 120 pg/mL (peak)  12 Hours ~ 110 pg/mL |
| 74 | Bai | 9 per group | C57BL/6 Mouse (8-10 weeks) | 8 Days | Day 0 - 5mL  Day 4- 3 mL | Day 7 - 3 mg, 1mL PBS | Pouch injected with 4 mL PBS and withdrawn | 6 Hours | Leukocytes 24 Hours ~ 10500/pouch | Not Measured |
| 75 | Albrase | 9 per group | C57BL/6 Mouse (8-10 weeks) | 8 Days | Day 0 - 3mL  Day 4- 3 mL | Day 7 - 3 mg, 1ml PBS | Pouch injected with 2 mL PBS containing 5 mM EDTA, massaged, and withdrawn | 6 Hours | Not measured | IL-1β   6 Hours ~ 110 pg/mL   TNF-α   6 Hours ~ 425 pg/mL |
| 76 | Du | 9 per group | C57BL/6 Mouse (8 weeks) | 7 Days | Day 0 - 3mL  Day 4- 3 mL | Day 6 - 2.5 mg, 1.1 ml PBS | Pouch injected with 3mL HBSS buffer and withdrawn twice. | 6 Hours | Leukocytes 6 Hour~ 12 ± 1 x 10^6^ /mL | Pouch Fluid Analyte Concentration   IL-1β   6 Hours ~ 1750 ± 500 pg /mL  CXCL-1  6 Hours ~ 2250 ± 750 pg /mL |
| 77 | Yang | At least 9 per group | Female and Male C57BL/6 Mouse (8-12 weeks) | 8 Days | Day 0 - 5 mL  Day 3 - 3 mL  Day 5 - 3 mL | Day 7 - 3 mg, 1 mL PBS | Animal euthanised, pouch dissected and washed with 2 mL PBS | 3, 6 12 Hours | Leukocytes 6 Hour~ 17.5 ± 2 x 10^5^ /mL | Not Measured |
| 78 | Jiang | 5 per group | Kunming Mouse 8-10 weeks) | 3 Days | Day 0 - 5 mL  Day 1 - 3 mL | Day 2 - 3 mg, 1 mL PBS | Technique not specified. | 5 Hours | Leukocytes   3 Hours ~ 2 ± 0.2 x10^6^ cells   6 Hours ~ 5.5 ± 0.5 x10^6^ cells   12 Hours ~ 11 ± 0.3 x 10^6^ cells   Macrophages   3 Hours ~ 1.5 ± 0.2 x10^6^ cells   6 Hours ~ 4.0 ± 0.8 x10^6^  cells   12 Hours ~ 5.5 ± 0.5 x 10^6^ cell  Neutrophils   3 Hours ~ 0.25 ± 0.1 x10^6^  cells   6 Hours ~ 1.0 ± 0.2 x10^6^ cells   12 Hours ~ 2.75 ± 0.25 x 10^6^ cell | IL-1β   3 Hours ~ 30 ± 10 pg/mL   6 Hours ~ 375 ± 75 pg/mL (peak)  12 Hours ~ 30 ± 10 pg/mL |
| 79 | Liao | Not specified | C57BL/6 Mouse (8-10 weeks) | 7 Days | Day 0 - 5 mL  Day 2 - 3 mL | Day 6 - 3 mg, 1 mL PBS | Pouch injected with 5 mL PBS, massaged, and withdrawn | 6 Hours | Not Measured | IL-1β   6 Hours ~ 220 ± 10 pg/mL |
| 80 | Li | 5 per group | C57BL/6 Mouse (8 weeks) | 6 Days | Day 0 – 5 mL  Day 3 – 5 mL | Day 6 – 3 mg, 1 mL PBS | Technique not specified. | 24 Hours | Neutrophils   6 Hours ~ 25 ± 2.5 % | IL-1β   6 Hours ~ 77.5 ± 2.5 pg/mL   TNF-α   6 Hours ~ 750 ± 25 pg/mL |
| 81 | Wu | 7 per group | C57BL/6 Mouse (age not specified ) | 7 Days | Day 0 - 5 mL  Day 3 - 3 mL | Day 6 - 3 mg, 1 mL saline | Animal euthanised, poch dissected, washed with 4 mL PBS | 6 Hours | Leukocytes 24 Hour~ 12 .5 ± 2.5 x 10^5^ /mL | IL-1β   24 Hours ~ 800 ± 50 pg/mL   IL-6   6 Hours ~ 20 ± 5 pg/mL |

‘Not measured’ denotes the variable was not an end point of the study, whereas ‘not presented’ indicates that although the variable was measured, the data was not presented in the study or supplementary materials. All animals used in studies were male unless explicitly stated. All counts or concentrations of leukocytes and analytes were expressed as mean ± standard deviation. The symbol ‘-’ indicates numerical data for a variable was presented in the study. The symbol ‘~’ signifies an approximation of the numerical data, visually determined from figures in the studies. ‘Peak’ denotes when peak cell infiltration, concentration or activity occurred. Considerable variability was noted for measurements and presentation of leukocyte counts. AU, absorbance unit; CCL, chemotactic ligand; CXC, chemokine (C-X-C motif) ligand; EDTA, Ethylenediaminetetraacetic acid; GADPH, Glyceraldehyde-3-phosphate dehydrogenase; GM-CSF, granulocyte macrophage colony-stimulating factor; HBSS, Hanks' Balanced Salt Solution; HIF, hypoxia inducible factor; hr, hour; IL, interleukin; IL-1Ra, interleukin-1 receptor antagonist; KC, keratinocyte chemoattractant; LT, Leukotriene; MCP, monocyte chemoattractant protein; mg, milligram; MIP, macrophage inflammatory protein; mL, millilitre; mm, millimetre; MPO, myeloperoxidase; mRNA, messenger ribonucleic acid ;mU, milliunit; ng, nanogram; nM, nanomolar; PBS, phosphate buffered saline; pg, picogram; PG, prostaglandin; PGF, platelet growth factor; PPIA Peptidylprolyl isomerase A; TNF, tumour necrosis factor; TX, thromboxane; µL, microlitre
