# Supplementary material for: Systematic review of the subcutaneous air pouch model using monosodium urate and calcium pyrophosphate and recommendations for studying crystal‐related arthropathies
Source: Animal Model Exp Med. 2025 Jul 11;8(9):1611–27. doi: 10.1002/ame2.70058 (PMC12531122; doi:10.1002/ame2.70058)
Supplement: Supplementary file 1 — Data S1. [file AME2-8-1611-s001.zip › Revision Feb 2025 Supplementary Material 2.docx]

**Supplementary Material**

**Table S2. Review of Methods for Generation and Performance of the Calcium Pyrophosphate Crystal Air Pouch Model and Endpoints**

| Entry Number | Author | Group Size | Animal (age) | Pouch Generation | | Pouch Stimulation Time Point, Number of Crystals and Suspension volume | Data Collection | | Analysis | |
| --- | --- | --- | --- | --- | --- | --- | --- | --- | --- | --- |
|  |  |  |  | Protocol Length | Air Pouch Inflation Time Points and volume |  | Pouch Fluid Harvesting Technique | Measurement Time Points (Post MSU delivery) | Cell Counts | Analytes |
| 1 | Brooks | 6 per group | Wistar Rat (age not specified) | 7-10 Days | Day 0 – 15 mL  Day 2 - 15 mL  Day 4 – 15 mL | Day 7-10 – 150 mg, 15 mL Saline (Administered via 18G dwelling catheter in pouch) | 1 mL aspirated via 18G indwelling catheter | 0,1,2,4,6, and 8 Hours | Rate of Leukocytes infiltration  0 Hours ~ 0.02 x 10^6^/ml/hr   1 Hours ~ 0.1 x 10^6^/ml/hr  2 Hours ~ 0.2 x 10^6^/ml/hr  4 Hours ~ 0.6 x 10^6^/ml/hr  6 Hours ~ 2.5 x 10^6^/ml/hr (Peak)  8 Hours ~ 0.1 x 10^6^/ml/hr | Rate of Plasma Extravasation   6 Hours~ 5.5. x 10-2 AU /hr (Peak)   LTB4  6 Hours~ 14 nM (Peak) |
| 2 | Sedgwick | >6 per group | Wistar Rat (Age not specified) | 2/4/7 Days | Day 0 – 20 mL Air Day 2-5 - 10 mL air given every 3 days to maintain inflation (criteria not specified) | Day 1 or 3 or 6 – 0.1 or 1 or 5 or 10 mg, 1 or 5 mL Saline | Pouch directly aspirated | 2, 4, 6 and 24 Hours | Leukocytes  6 Day Pouch, 4 Hours, 0.1 mg Crystals  1 mL suspension volume ~ 5 x 10^6^ cells  5 mL suspension volume ~ 10 x 10^6^ cells  6 Day Pouch, 4 Hour,1.0 mg Crystals  1 mL suspension volume ~ 4 x 10^6^ cells  5 mL suspension volume ~ 10 x 10^6^ cells  6 Day Pouch, 4 Hour, 5.0 mg Crystals  1 mL suspension volume ~ 6 x 10^6^ cells  5 mL suspension volume ~ 17.5 x 10^6^ cells  6 Day Pouch, 4 Hour,10.0 mg Crystals  20 mL suspension volume ~ 3 x 10^6^ cells  5 mL suspension volume ~ 50 x 10^6^ cells  1 Day pouch, 10 mg Crystals in 5 mL  2 Hour~ 10 x 10^6^ cells  4 Hour~ 10 x 106 cells  6 Hour~ 10 x 10^6^ cells  24 Hour~ 10 x 10^6^ cells  3 Day pouch, 10 mg Crystals in 5 mL  2 Hour~ 10 x 10^6^ cells  4 Hour~ 25 x 10^6^ cells  6 Hour~ 25 x 10^6^ cells  24 Hour~ 10 x 10^6^ cells  6 Day pouch, 10 mg Crystals in 5 mL  2 Hour~ 10 x 10^6^ cells  4 Hour~ 75 x 10^6^ cells  6 Hour~ 90 x 10^6^ cells  24 Hour~ 80 x 10^6^ cells | Not measured |
| 3 | Torres | 5 per group | C57BL/6 (12-16 weeks) | 7 Days | Day 0 – 5 mL  Day 3 – 3 mL | Day 7 - 1 mg, 0.5 mL PBS | 5 mL stain buffer injected into pouch and withdrawn | 6 Hours | Neutrophils   6 Hours ~ 3.5 x 10^6^ /pouch | Not measured |
| 4 | Laure Campillo-Gimenez | Not specified | C57BL/6J Mice (8 Weeks) | 7 Days | Day 0 – 3ml Day 3 – 3ml | Day 6 - 1 mg/mL in PBS (amount of crystals and suspension volume not specified) | Animal sacrificed, pouch dissected and washed twice with 2 mL PBS | 6 and 24 Hours | m-CPP Leukocytes   6 Hours ~ 7 x 10^6^ /pouch, Neutrophils: 86 ± 9%)  24 Hours ~ 4 x 10^6^ /pouch Neutrophils: 63 ± 2%)  t-CPP Leukocytes   6 Hours ~ 1.5 x 106 /pouch, Neutrophils: 59 ± 4%)  24 Hours ~ 0.5 x 106 /pouch Neutrophils: 31 ± 3%) | m-CPP IL-1β   6 Hour~ 70 pg/mL  24Hour~ 10 pg/mL  CXCL-1-   6 Hours~ 175 pg/mL  24 Hours ~ 10 pg /mL   t-CPP IL-1β   6 Hours ~ 5 pg/mL  24Hours ~ 0 pg/mL  CXCL-1-   6 Hours ~ 20 pg/mL  24 Hours ~ 0 pg /mL |
| 5 | Gordon | 4 per group | Wistar (age not specified) | 8 Days | Day 0 – 10 mL  Day 2 – 10 mL Day 5 - 10 mL | Day 6 – 50 mg, 5 mL PBS | Animal sacrificed, pouch dissected. No pouch fluid collected. | 48 Hours | Not measured | Not measured |
| 6 | Hemstapat | 6 per group | Wistar rat (7-8 weeks) | 7 Days | Day 0 - 20 mL  Day 3 - 10 mL | Day 6 - 15 mg, 10 mL HEPES buffered saline | Technique not specified. | 24 Hours | Leukocytes 24 Hour~ 10500/pouch | Not Measured |
| 7 | Kumagai | 5 per group | Wistar rat (7 weeks) | 9 Days | Day 0 - 20 mL  Day 1 - 10 mL | Day 7 - 5mg, 10 mL saline | Animal sacrificed, pouch dissected and washed twice with 5 mL PBS | 0,6,9,12,24 and 48 Hours. | Leukocytes  0 Hours ~ 2 x 10^6^ cells  6 Hours ~ 25 x 10^6^ cells   9 Hours ~ 27.5 x 10^6^ cells   12 Hours ~ 29 x 10^6^ cells (peak)   24 Hours ~ 25 x 10^6^ cells   48 Hours ~ 20 x 10^6^ cells | PGE_2_   0 Hours ~ 0 ng/mL  6 Hours ~ 57.5 ng/mL (peak)   9 Hours ~ 50 ng/mL  12 Hours ~ 40 ng/mL  24 Hours ~ 5 ng/mL  48 Hours ~ 1 ng/mL |
| 8 | Sin | >5 per group | Wistar rat (age not specified) | 7Days | Day 0 – 10 mL  Day 2 – 10 mL Day 5 - 10 mL | Day 5 - Crystal suspension composition not specified | Technique not specified. | 2,4,6,24 Hours | Leukocytes   2 Hours ~ 10 x 10^6^ cells   4 Hours ~ 50 x 10^6^ cells   6 Hours ~ 120 x 10^6^ cells (peak)   24 Hours ~ 110 x 10^6^ cells | Not Measured |

‘Not measured’ denotes the variable was not an end point of the study, whereas ‘not presented’ indicates that although the variable was measured, the data was not presented in the study or supplementary materials. All animals used in studies were male. All counts or concentrations of leukocytes and analytes were expressed as mean ± standard deviation. The symbol ‘–’ indicates numerical data for a variable was presented in the study. The symbol ‘~’ signifies an approximation of the numerical data, visually determined from figures in the studies. ‘Peak’ denotes when peak cell infiltration, concentration or activity occurred. Considerable variability was noted for measurements and presentation of leukocyte counts. AU, absorbance unit; CXC, chemokine (C-X-C motif) ligand; EDTA, Ethylenediaminetetraacetic acid; HEPES BSS, 4-(2-hydroxyethyl)-1-piperazineethanesulfonic; hr, hour; IL, interleukin; LT, Leukotriene; mL, millilitre; PBS, phosphate buffered saline; pg, picogram; PG, prostaglandin; TNF, tumour necrosis factor; µL, microlitre
