# Supplementary material for: Systematic review of the subcutaneous air pouch model using monosodium urate and calcium pyrophosphate and recommendations for studying crystal‐related arthropathies
Source: Animal Model Exp Med. 2025 Jul 11;8(9):1611–27. doi: 10.1002/ame2.70058 (PMC12531122; doi:10.1002/ame2.70058)
Supplement: Supplementary file 1 — Data S1. [file AME2-8-1611-s001.zip › Supplementary Material Protocol S2.docx]

**Summarised Protocol**

**Materials**

C57BL/6 Mice

Inhalation Aesthetic 5% v/v isoflurane (in 100% O_2_)

70% (v/v) ethanol

0.9% saline, sterile

PBS, sterile

Relevant kits for sample analysis – ELISA kits, biochemistry assays

Haemocytometer

Light microscope

Differential WBC stanning kit

MSU and CPP crystals (additional equipment required for synthesis)

Sonicator

Reagents for tissue fixation and H&E staining

Test compounds, dexamethasone

EDTA

5, 10 mL and 20 mL syringes, sterile

27-, 25- and 21-G needles, sterile

0.2-μm syringe filter

Anaesthetic chamber

Equipment for animal euthanisation

Animal hair clipper

Forceps and scissors

Centrifuge tubes

Transfer pipettes, sterile

Equipment for storage of samples

**Preparation of Animals**

1. Acclimatise animals at constant temperature, under 12hr light-dark cycle and with food and water *ad libitum.*
2. Allocate at least 6 animals for each group consisting of vehicle, sham, positive control and experimental conditions.
3. Ensure appropriate MSU and/or CPP crystal preparations are purchased or synthesised using optimised published methods, confirmed under light microscopy, dimensions quantified using scanning electron microscopy and confirmed to be endotoxin free using endotoxin assay kit.
4. Ensure appropriate studies have been completed to determine peak concentration time of therapy to be administered.
5. Ensure appropriate studies have been completed to assess the peak timepoints for meditators to be assessed.
6. Ensure appropriate studies have been completed to optimise straining protocols for tissue analysis.

**Generation of Subcutaneous Air Pouch**

1. On Day 0, draw room air into a syringe (Rat: 20 mL syringe , Mouse 10 mL syringe), attach a 0.2-μm sterile filter to the syringe and then a 27-G needle to the filter output.
2. Anaesthetise the animal under 4-5 % isoflurane for induction and 1-2% isoflurane for maintenance, at 1 L/min in 100% oxygen. Once the animal is unconscious, shave the dorsal surface around the area of the scapula (Rat: 3 x 3 cm, Mouse: 1.5 x 1.5 cm) and sterilise the region with 70% ethanol.
3. Insert the needle 5 mm into the skin within the invagination, and deliver air slowly (Rat:20 mL, Mouse:5 mL) into the subcutaneous space while placing a second researchers fingers laterally below where the pouch is to be inflated to ensure a single cavity is formed. Upon needle removal, pinch the puncture site for 20 seconds, return the animal to the cage and observe until they recover.
4. On Day 4, anaesthetise the animal again, sterilise with 70% ethanol and deliver an additional volume of air (Rat:10 mL, Mouse:3 mL) using the same method.

**Pre-treatment of Animals**

1. On Day 7, pre-treat the animals with the test compound via the desired route. The time of injection of the test compound is based on its pharmacokinetic profile. The vehicle and sham group are pre-treated with an identical volume of vehicle. A positive control group is pre-treated with prednisolone 5 mg/kg i.p. 30 min prior to crystal delivery.

**Induction of Inflammation**

1. On Day 7, suspend MSU crystals (Rat:15 mg of crystals in 5 mL saline, Mouse: 3 mg crystal in 1 mL PBS) or CPP crystals (Rat: 30 mg of crystals in 10 mL saline, Mouse: 1 mg crystal in 0.5 mL PBS) and after sonicating for 10 min, draw the crystals into a 5 mL syringe. Attach a 21 G needle to the syringe.
2. Anaesthetise the animal, once unconscious, with the animal on their side, slowly deliver the crystal suspension into the pouch from the same direction as the air inflation. For the sham group, deliver 5 mL saline in the Rat and 1 mL of PBS in the Mouse without crystals. Upon needle removal, pinch the insertion point and gently massage the pouch cavity for 1 minute.

**Harvesting pouch fluid**

1. 6 hours after induction of inflammation, anaesthetise the animal and once unconscious place the animal on their side.
2. Draw sterile 5mL saline (Rat) or 2 mL PBS (Mouse) into a 5 ml syringe and attach a 21 G needle. Inject the saline into the pouch cavity where the exudate accumulates and gently massage for 1 minute. Withdraw the fluid (Rat:5 mL, Mouse:2 mL) from the pouch. If encountering resistance during withdrawal, whilst massaging the pouch, gently reinfuse and withdraw fluid until the volume is collected. Void further attempts if the collection proves difficult.
3. Repeat fluid collections at 12, 24 and 48 hours using the same technique at different sites on the pouch.

**Cell Count and Inflammatory Mediator Analysis**

1. Maintain the pouch fluid on ice during sample handling. Add EDTA to the pouch fluid to obtain a solution containing 5.4 mM EDTA.
2. Remove cells from pouch fluid via centrifugation (1000 x *g*, 8 min, 4^0^C). Divide the supernatant into 0.5 mL aliquots and freeze in -80^o^C for analysis of inflammatory mediators using commercially available ELISA kits or biochemistry assays.
3. Resuspend the cell pallet in 0.5mL ice-cold PBS and perform a manual total cell count using trypan blue and a differential cell count using differential staining kit.

**Pouch tissue analysis**

1. Euthanise animals using CO_2_ asphyxiation and place the animal prone. Using scissors, expose the apex of the pouch membrane by generating a subcutaneous T-shaped incision into the dorsal skin overlying the pouch. Separate the overlying skin and adjacent subcutaneous and paraspinal tissue by blunt dissection. Grasp and elevate the pouch membrane with forceps and cut at the base with scissors.
2. Fix the isolated membrane in 10% paraformaldehyde, embed in paraffin, section in 5 μm cuts and stain with optimised H&E protocol. Assess inflammation within the pouch tissue using the scoring system outlined in **Table 1,** created by Huang et al.

**Table 1. Histological Scoring System for Quantifying Inflammation in the Air Pouch Model.**[1]

|  | Cell Types | Score | | | | |
| --- | --- | --- | --- | --- | --- | --- |
|  |  | 0 | 1 | 2 | 3 | 4 |
| Inflammation | Polymorphonuclear  cells (neutrophils  and eosinophils) | None | Minimal,  1-5/per  HPF | Slight,  6-15/per  HPF | Moderate,  16-25/per  HPF | Severe,  >25/per  HPF |
|  | Mononuclear cells  (lymphocytes and  macrophages) | None | Minimal,  1-5/per  HPF | Slight,  6-15/per  HPF | Moderate,  16-25/per  HPF | Severe,  >25/per  HPF |
| Membrane thickness of murine  air pouch | | None | <50 μm | 50-75 μm | 76-100 μm | >100 μm |

HPF, High Power Field (40x); μm, micrometer. The severity of inflammation is graded based on the sum of individual scores for polymorphonuclear and mononuclear cell infiltration.

**References**

1. 1. Huang JH, Chiang BL. Regulatory T cells induced by B cells suppress NLRP3 inflammasome activation and alleviate monosodium urate-induced gouty inflammation. iScience. 2021;24(2):102103.
